# Genetic and Transcriptional Regulatory Mechanisms of Lipase Activity in the Plant Pathogenic Fungus Fusarium graminearum

**DOI:** 10.1128/spectrum.05285-22

**Published:** 2023-04-24

**Authors:** Sieun Kim, Juno Lee, Jiyeun Park, Soyoung Choi, Duc-Cuong Bui, Jung-Eun Kim, Jiyoung Shin, Hun Kim, Gyung Ja Choi, Yin-Won Lee, Pahn-Shick Chang, Hokyoung Son

**Affiliations:** a Department of Agricultural Biotechnology, Seoul National University, Seoul, Republic of Korea; b Department of Pathology, University of Texas Medical Branch, Galveston, Texas, USA; c Research Institute of Climate Change and Agriculture, National Institute of Horticultural and Herbal Science, Jeju, Republic of Korea; d Division of Bioresources Bank, Honam National Institute of Biological Resources, Mokpo, Republic of Korea; e Center for Eco-friendly New Materials, Korea Research Institute of Chemical Technology, Daejeon, Republic of Korea; f Research Institute of Agriculture and Life Sciences, Seoul National University, Seoul, Republic of Korea; g Center for Food and Bioconvergence, Seoul National University, Seoul, Republic of Korea; h Center for Agricultural Microorganism and Enzyme, Seoul National University, Seoul, Republic of Korea; Beijing Forestry University

**Keywords:** *Fusarium graminearum*, lipases, transcription factors

## Abstract

Lipases, which catalyze the hydrolysis of long-chain triglycerides, diglycerides, and monoglycerides into free fatty acids and glycerol, participate in various biological pathways in fungi. In this study, we examined the biological functions and regulatory mechanisms of fungal lipases via two approaches. First, we performed a systemic functional characterization of 86 putative lipase-encoding genes in the plant-pathogenic fungus Fusarium graminearum. The phenotypes were assayed for vegetative growth, asexual and sexual reproduction, stress responses, pathogenicity, mycotoxin production, and lipase activity. Most mutants were normal in the assessed phenotypes, implying overlapping roles for lipases in F. graminearum. In particular, FgLip1 and Fgl1 were revealed as core extracellular lipases in F. graminearum. Second, we examined the lipase activity of previously constructed transcription factor (TF) mutants of F. graminearum and identified three TFs and one histone acetyltransferase that significantly affect lipase activity. The relative transcript levels of *FgLIP1* and *FGL1* were markedly reduced or enhanced in these TF mutants. Among them, Gzzc258 was identified as a key lipase regulator that is also involved in the induction of lipase activity during sexual reproduction. To our knowledge, this study is the first comprehensive functional analysis of fungal lipases and provides significant insights into the genetic and regulatory mechanisms underlying lipases in fungi.

**IMPORTANCE** Fusarium graminearum is an economically important plant-pathogenic fungus that causes Fusarium head blight (FHB) on wheat and barley. Here, we constructed a gene knockout mutant library of 86 putative lipase-encoding genes and established a comprehensive phenotypic database of the mutants. Among them, we found that FgLip1 and Fgl1 act as core extracellular lipases in this pathogen. Moreover, several putative transcription factors (TFs) that regulate the lipase activities in F. graminearum were identified. The disruption mutants of F. graminearum-lipase regulatory TFs all showed defects in sexual reproduction, which implies a strong relationship between sexual development and lipase activity in this fungus. These findings provide valuable insights into the genetic mechanisms regulating lipase activity as well as its importance to the developmental stages of this plant-pathogenic fungus.

## INTRODUCTION

The filamentous ascomycete fungus Fusarium graminearum is a causal agent of Fusarium head blight (FHB) on wheat and barley as well as ear rot on maize (1). F. graminearum infection leads to significant yield losses and mycotoxin contamination (e.g., deoxynivalenol [DON] and zearalenone [ZEA]) on the infected grains, which threatens food and feed safety (2, 3). To understand the molecular mechanisms underlying various biological processes as well as the virulence of F. graminearum, a number of studies have been conducted through large-scale reverse genetic approaches (4–11). However, major outbreaks of FHB continue to occur (12, 13), and studies on fungal development and pathogenicity are still needed.

Lipases (EC 3.1.1.3) are a class of carboxyl ester hydrolases (EC 3.1.1.-) that break the ester linkages of long-chain triglycerides, diglycerides, and monoglycerides and produce free fatty acids and glycerol, whereas “true” esterases (EC 3.1.1.1) catalyze the hydrolysis of simple esters and short-chain fatty acids (14). These enzymes also catalyze the synthesis and transesterification of the ester bonds to generate new esters (15). Fungal lipases are mostly extracellular and are significantly induced by environmental factors, such as nitrogen and carbon sources, especially in the presence of a lipid source (15, 16). Besides the utilization of extracellular lipid sources, fungal lipases participate in various biological processes, such as appressorium formation (17), autophagy (18, 19), cell cycle progression (20), and membrane lipid synthesis (21).

Plant-pathogenic fungi secrete diverse extracellular enzymes during infection, such as cutinases and lipases, to degrade plant cuticle layers, which are composed of waxes and lipid polymers called cutin (22–24). Previous studies of Botrytis cinerea and Alternaria brassicicola showed that the addition of antilipase antibodies to a conidial suspension led to reduced lesion formation (25, 26). The pretreatment of wheat leaves with Lip1 resulted in reduced epicuticular wax crystalloids on the leaf surfaces in Blumeria graminis (27). In F. graminearum, the secreted lipase Fgl1 has been characterized as a virulence factor that represses the vascular callose deposition of the wheat by releasing free fatty acids (24, 28). Another secreted lipase, namely, FgLip1, and several feruloyl esterases in F. graminearum were reported to be dispensable for pathogenicity (29, 30). Lipase mutants in Fusarium oxysporum, *B. cinerea*, and Magnaporthe oryzae also retained full virulence on the host plants (31–33).

In F. graminearum, several studies have reported the importance of lipid metabolism in the sexual developmental stages. Gene expression profiling suggested that lipid biosynthesis occurs in early sexual development and that it is followed by lipid oxidation processes (34, 35). Palmitoyl-2-oleoyl-3-linoleoyl-rac-glycerol was reported to be a major lipid that is required for sexual development in this fungus (36). Also, Fpo1, which is a negative regulator of perithecium development in F. graminearum, was reported to regulate fatty acid metabolism, and the mutant showed enhanced perithecium formation with excess lipid accumulation during sexual development (37).

The aims of this study were (i) to functionally characterize putative lipase-encoding genes for vegetative growth, asexual and sexual reproduction, stress response, pathogenicity, mycotoxin production, and lipase activity, as well as (ii) to identify transcription factors (TFs) regulating the lipase activity in F. graminearum. In this study, we constructed a genome-wide knockout mutant library of 86 putative lipase-encoding genes in F. graminearum and functionally characterized all of the mutants. We identified three TFs and one histone acetyltransferase regulating the lipase activity by screening the extracellular lipase activity of previously constructed TF mutants (4). We also demonstrated the relationship between sexual reproduction and lipase activity in F. graminearum. Our findings and genetic resources provide insights into the genetic and regulatory mechanisms of the lipase activity underlying fungal development.

## RESULTS

### Identification and deletion of putative lipase-encoding genes in F. graminearum.

We identified putative lipases in F. graminearum, based on conserved lipase and esterase domains, using InterProScan (38) and Pfam (39). We manually added 9 putative lipases, based on the MIPS F. graminearum genome database (40). A total of 86 putative lipases were identified and classified based on Pfam terms (41) and an NCBI Conserved Domains Database (CDD) search (42) (Table 1; Table S1), in which 57% of them belong to the α/β hydrolase fold enzymes, 19% of them belong to the SGNH hydrolases, and the rest of them belong to families with phospholipase domains.

**TABLE 1:** Classification of putative lipases in F. graminearum

Superfamily	Family	Description	No.
Abhydrolase	Lipase_3	Lipase (class 3)	5
	LIP	Secretory lipase	3
	Abhydro_lipase	Partial alpha/beta-hydrolase lipase region	1
	Abhydrolase_1	alpha/beta hydrolase fold	1
	Abhydrolase_2	Phospholipase/Carboxylesterase	5
	Abhydrolase_3	Alpha/beta hydrolase fold	7
	Coesterase	Carboxylesterase	16
	Tannase	Tannase and feruloyl esterase	6
	DUF676	Putative serine esterase (DUF676)	4
	PGAP1	PGAP1-like protein	1
SGNH_hydrolase	Lipase_GDSL	GDSL-like lipase/Acylhydrolase	3
	Lipase_GDSL_2	GDSL-like lipase/Acylhydrolase	13
Patatin_and_cPLA2	Patatin	Patatin-like phospholipase	10
Phospholip_A2_3	Phospholip_A2_3	Prokaryotic phospholipase A2	1
PLA2_B	PLA2_B	Lysophospholipase catalytic domain	6
PLDc	PLDc	Phospholipase D Active site motif	2
	PLDc_2	PLD-like domain	2
Total			86

To investigate the biological functions of these lipases in F. graminearum, we disrupted all 86 putative lipase-encoding genes via homologous recombination and the split-marker method. The open reading frame (ORF) of each gene was replaced with a Geneticin resistance gene cassette (*GEN*), and the transformants were screened via Geneticin resistance and diagnostic PCR. The deletion mutants were further confirmed via Southern blotting hybridization (Fig. S1). We characterized phenotypic changes in the vegetative growth, conidiation, sexual reproduction, stress response, mycotoxin production, virulence, and lipase activity for the deletion mutants. Most mutants had no defects in the assessed phenotypes, which implies a significant functional redundancy of lipases in F. graminearum. The phenotypic data are summarized in Table S1.

### Vegetative growth, conidiation, and sexual development.

**FIG 1:**
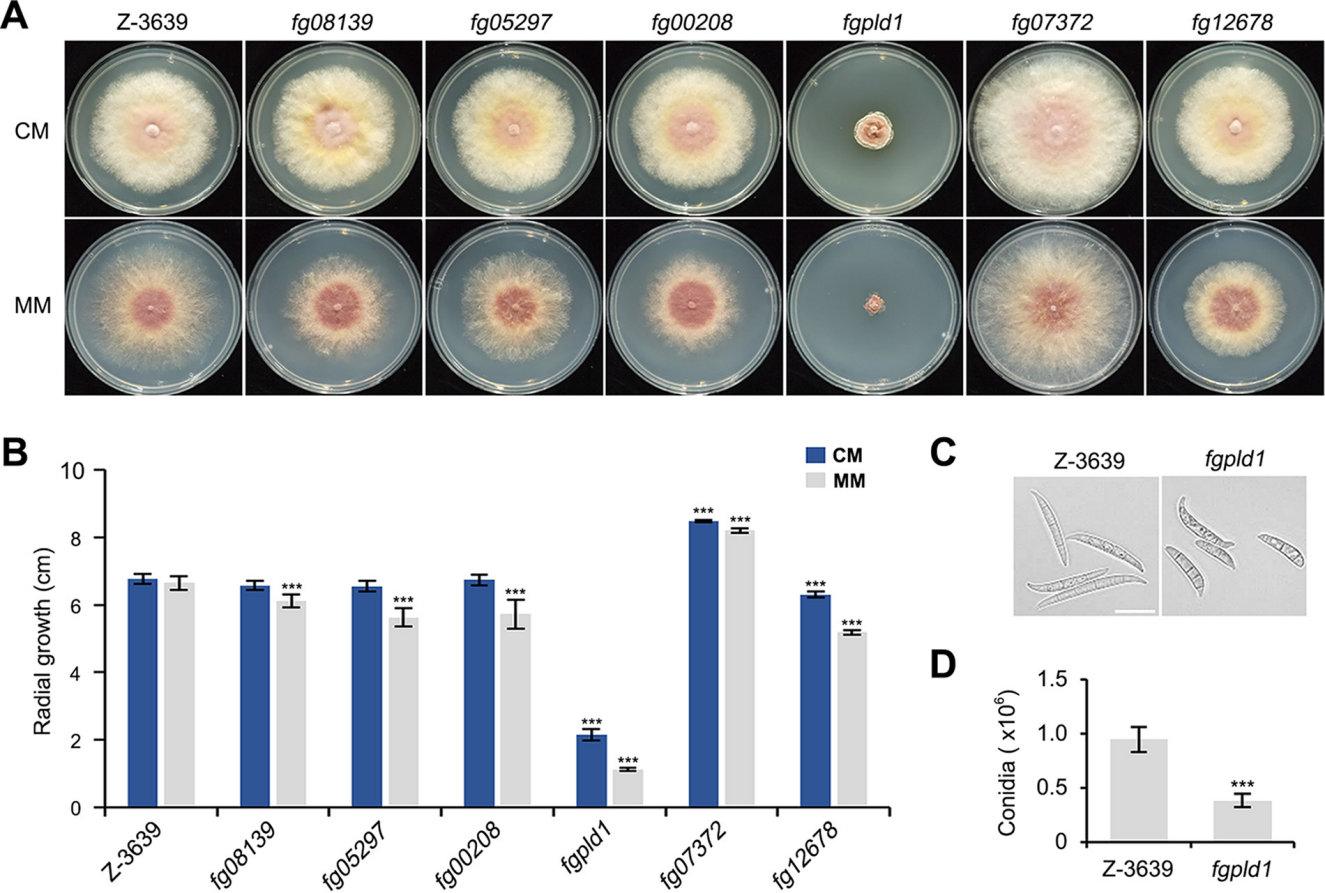
Vegetative growth and conidiation of lipase mutants. (A) The mycelial growth on complete medium (CM) and minimal medium (MM). The strains were imaged 4 days after inoculation. (B) Radial growth on CM and MM. Radial growth was measured 4 days after inoculation. Significant differences (***, *P* < 0.001), compared to the wild-type, are indicated by asterisks. (C) Conidial morphology. The photographs were taken 3 days after conidial induction in carboxymethyl cellulose medium (CMC). Scale bar = 20 μm. (D) Conidial production. The number of conidia was measured 5 days after inoculation in CMC.

We observed the vegetative growth and colony morphology of the mutants on complete medium (CM) and minimal medium (MM). *fg08139*, *fg05297*, *fg00208*, *fgpld1*, and *fg12678* showed reduced mycelial growth, especially on MM, and, interestingly, the growth rate of *fg07372* was markedly enhanced, compared to the wild-type, on both CM and MM (Fig. 1A and B).

To assess conidiation, we counted the number of conidia and observed the conidial morphology of the mutants in carboxymethyl cellulose medium (CMC). Only *fgpld1* showed reduced conidiation (by approximately 65%) and produced abnormal conidia with shorter sizes, as described in a prior study (43) (Fig. 1C and D).

**FIG 2:**
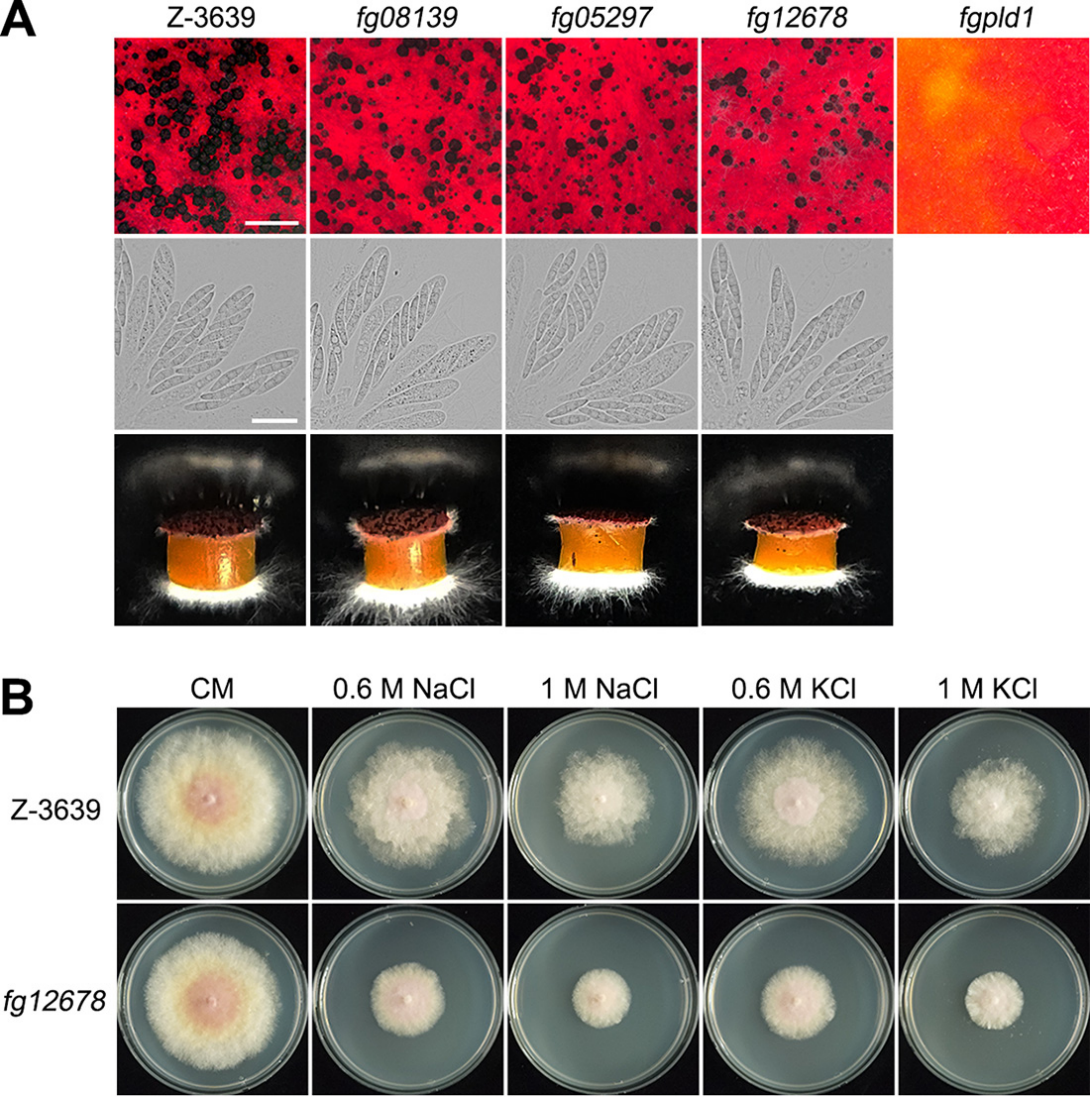
Sexual development and stress response. (A) Perithecium formation, ascospore formation, and forcible ascospore discharge. The perithecia and the asci were imaged 8 days after sexual induction on carrot agar. Scale bar = 1,000 μm (top panel, for dissecting microscopic pictures), 20 μm (middle panel for bright field [BF] images). The white cloudy materials (bottom panel) show the discharged ascospores. The discharged ascospores were imaged 48 h after the initiation of the assay. (B) Osmotic stress sensitivity. Strains were incubated on CM and on CM supplemented with osmotic stress agents (0.6 M NaCl, 1 M NaCl, 0.6 M KCl, 1 M KCl). The photographs were taken 5 days after inoculation.

With respect to sexual development, *fg08139*, *fg05297*, *fgpld1*, and *fg12678*, which showed reduced mycelial growth, were also defective in perithecial maturation. *fg08139*, *fg05297*, and *fg12678* produced a number of smaller immature perithecia than did the wild-type, but no mutant had any defects in the morphology of the ascospores or in ascospore discharge (Fig. 2A). The deletion of *fgpld1* completely abolished perithecial formation, as shown in the previous study (43).

### Stress response, mycotoxin biosynthesis, and virulence.

We evaluated the responses of the mutants under various stress conditions, including oxidative stress, osmotic stress, cell wall stress, pH stress, and several fungicide treatments. A few mutants had altered sensitivity or resistance to the stress agents (Table S1). Among them, *fg12678* exhibited strong sensitivity to osmotic stress induced by NaCl and KCl (Fig. 2B).

When assayed for mycotoxin production, the amounts of zearalenone (ZEA) and deoxynivalenol (DON) were enhanced in *fgpld1* (Table S1). For virulence, only *fgpld1* showed defects in virulence, as was previously reported (43).

These phenotypic results indicate that some lipases and esterases are involved in the important biological processes but that most play redundant roles in F. graminearum so that the loss of a single gene does not lead to significant phenotypic defects.

### FgLip1 and Fgl1 act as core extracellular lipases in F. graminearum.

**FIG 3:**
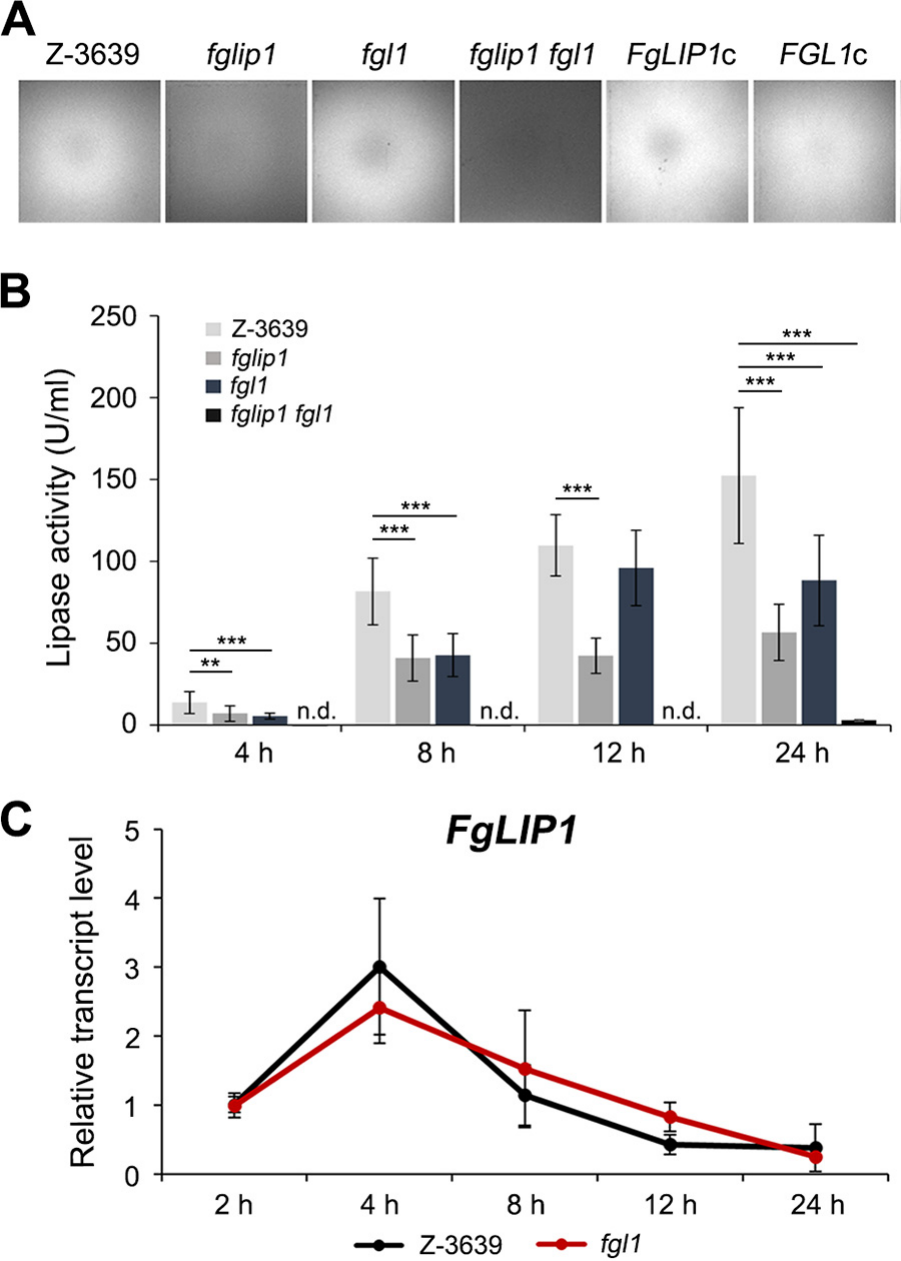
Lipase activity of *fglip1* and *fgl1*. (A) Lipase activity of *fglip1*, *fgl1*, *fglip1 fgl1*, and the complemented strains. Each strain was inoculated on MM supplemented with 1% olive oil and 0.0005% rhodamine B. Images were captured 2 days after inoculation under UV light. (B) Lipase activity of *fglip1*, *fgl1*, and *fglip1 fgl1.* The culture supernatant of each strain was harvested at the indicated time points after inoculation in the lipase-inducing medium. Lipase activity was measured spectrophotometrically, using para-nitrophenyl palmitate (pNPP) as a substrate. An asterisk indicates a significant difference (**, *P* < 0.005; ***, *P* < 0.001) between the wild-type and the deletion mutant at each time point. n.d., not detected. (C) Fold changes of *FgLIP1* in *fgl1.* Total RNA was extracted after 2, 4, 8, 12, and 24 h of cultivation in the lipase-inducing medium. The transcript abundances were estimated via qRT-PCR.

Olive oil is primarily composed of triglycerides (approximately 99%) and secondarily composed of other lipid sources (44). To evaluate the lipase activity, we inoculated the mutants on MM supplemented with olive oil as a sole carbon source, which was used as a lipase-inducing medium for the overall experiments. The fluorescence formed by the interaction of rhodamine B and free fatty acids was observed 2 days after inoculation under ultraviolet (UV) light. Contrary to expectations, no mutants exhibited reduced fluorescence, except for the deletion mutant of *FgLIP1*, which was previously reported to encode a secreted lipase (29) (Fig. 3A). The interesting thing was that the deletion of *FGL1*, which led to significantly reduced lipase activity in a prior study (24), made no difference to the fluorescence. We hypothesized that FgLip1 might primarily act on the hydrolysis of triglycerides, which compose the majority of olive oil, whereas Fgl1 might have different substrate preferences.

In order to precisely analyze the lipase activities of *fglip1* and *fgl1*, we took two approaches. First, we constructed a *fglip1 fgl1* double mutant as well as the complemented strains of the mutants. *fglip1 fgl1* exhibited almost no fluorescence, whereas weak fluorescence was still detected in *fglip1* (Fig. 3A). The fluorescence was restored in the *FgLIP1* complemented strain. Second, we cultured the strains in the lipase-inducing medium, and the lipase activities of the culture supernatants were measured at various time points, using para-nitrophenyl palmitate (pNPP) as a substrate (Fig. 3B). The amount of para-nitrophenol released by the enzymatic hydrolysis of pNPP was measured spectrophotometrically (45). The lipase activity of the wild-type increased over time after induction, and *fglip1* had consistently reduced activity, compared with the wild-type. *fgl1* showed reduced lipase activity within the first 8 h, but it almost recovered its activity at 12 h after inoculation (Fig. 3B). The lipase activity of *fglip1 fgl1* either was not detected or was barely detected at the overall time points, indicating that the extracellular lipase activity of F. graminearum completely depends on FgLip1 and Fgl1. To figure out whether the recovery of the lipase activity in *fgl1* at 12 h was due to the upregulation of *FgLIP1*, we analyzed the relative transcript level of *FgLIP1* at 2, 4, 8, 12, and 24 h after inoculation in the lipase-inducing medium via quantitative reverse transcription (qRT)-PCR assays (Fig. 3C). The wild-type and *fgl1* showed similar expression patterns of *FgLIP1*, which implies that a mechanism other than transcriptional regulation exists to regulate the lipase activity.

**FIG 4:**
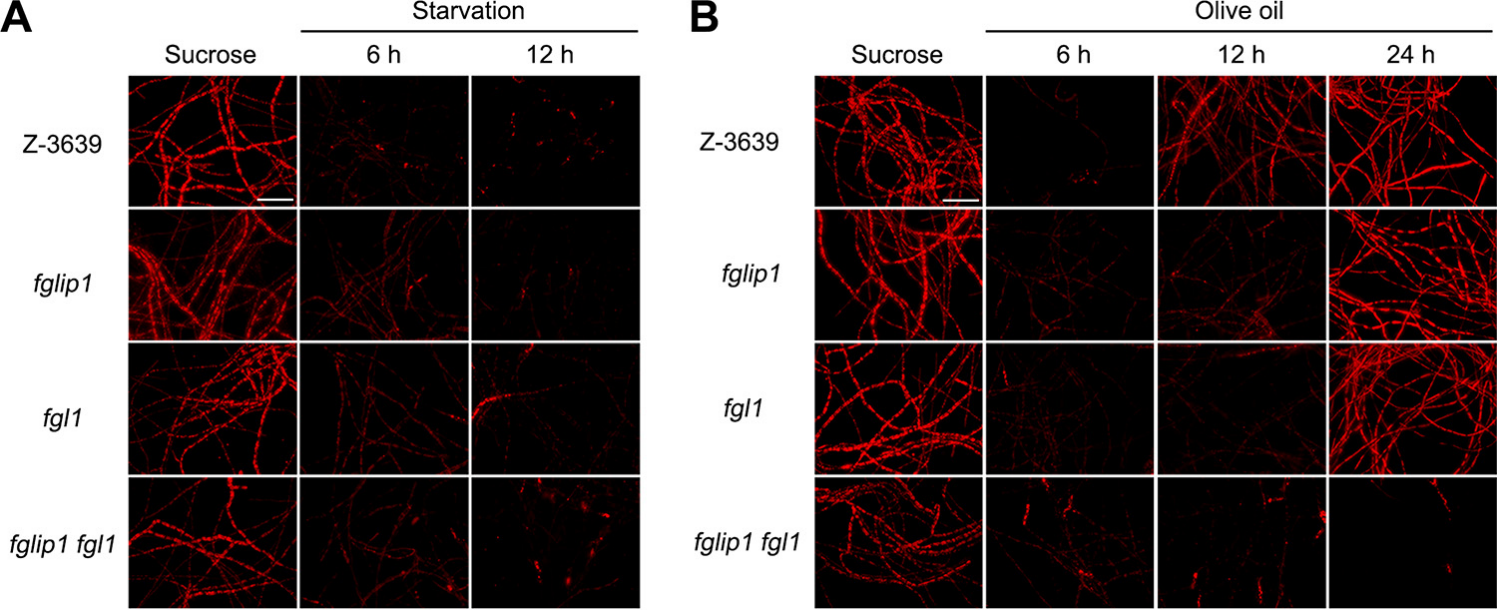
Visualization of intracellular lipid droplets in lipase mutants. (A and B) The mycelia were cultured in MM, MM without a carbon source (A), and MM supplemented with 1% olive oil as a sole carbon source (B) for the indicated amounts of time. The mycelia were stained with Nile red solution to observe intracellular lipid droplets. Scale bar = 50 μm.

Previous studies showed that F. graminearum accumulates lipid droplets when cultured in a rich medium and degrades the stored lipid droplets under carbon starvation conditions (19, 46). We hypothesized that the extracellular lipase activity of the strains might also affect the distribution of the intracellular lipid droplets under starvation or olive oil-supplemented conditions. We observed the distribution of intracellular lipid droplets via Nile red staining to investigate the lipid droplet utilization and accumulation of the mutants. All of the strains grew normally and accumulated lipid droplets in the mycelia when sucrose was given. Under starvation conditions, the lipid droplets significantly decreased in all strains (Fig. 4A). When olive oil was supplemented as the sole carbon source, the lipid droplets decreased after 6 h of cultivation (Fig. 4B). However, after 12 h, the wild-type recovered the intracellular lipid droplets by normally utilizing the olive oil as a carbon source. *fglip1* and *fgl1* showed a delay in lipid droplet recovery but started to accumulate the lipid droplets 24 h after inoculation. *fglip1 fgl1* failed to recover the intracellular lipid droplets after 24 h, which corresponds to the barely detected lipase activity in Fig. 3B.

These results suggest that the extracellular lipase activity in F. graminearum depends entirely on FgLip1 and Fgl1 and that the absence of both *FgLIP1* and *FGL1* almost completely blocks the extracellular lipase activity of F. graminearum.

### The enzymatic characteristics of FgLip1 and Fgl1.

**FIG 5:**
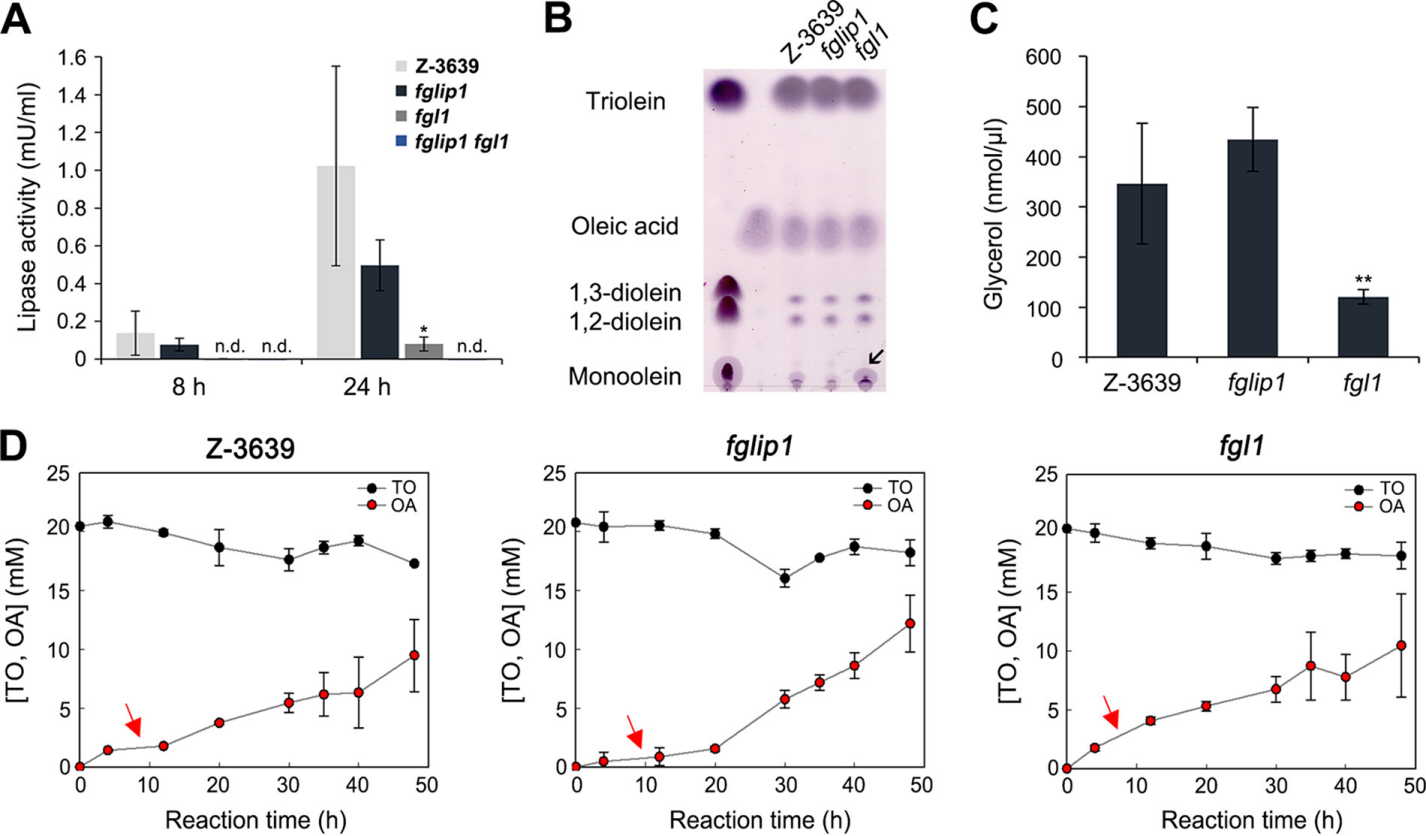
Enzymatic characteristics of FgLip1 and Fgl1. (A) Triacylglycerol lipase activity. The strains were incubated in the lipase-inducing medium for the indicated amounts of time. The culture supernatants were harvested and used for an analysis of the triacylglycerol lipase activity. An asterisk indicates a significant difference (*, *P* < 0.05) between the wild-type and the deletion mutant at each time point. n.d., not detected. (B) Thin-layer chromatography (TLC) of the hydrolysis products of triolein. 40 U of culture supernatants were reacted with 10 μL of triolein at 37°C for 20 h on a rotary shaker. Total lipids were extracted from the reaction mixture and compared with reference standards on the TLC plates: oleic acids, monoolein, 1,2-diolein, 1,3-diolein, and triolein. The arrow indicates the accumulated monoolein. (C) Glycerol assay. The amount of glycerol in the reaction mixture was measured. An asterisk indicates a significant difference (**, *P* < 0.005), compared to the wild-type. (D) Quantification of triolein and oleic acids. 50 U of culture supernatants were reacted with 40 mM triolein dissolved in isooctane at 37°C with vigorous mixing. Total lipids were extracted at 0, 4, 12, 20, 30, 35, 40, and 48 h after the reaction, and a portion of the lipid extracts was used to analyze the amounts of triolein and oleic acid via high-performance liquid chromatography (HPLC). The arrow indicates the amount of oleic acid produced at the beginning of the reaction. TO, triolein; OA, oleic acid.

To further investigate the enzymatic characteristics of FgLip1 and Fgl1, we analyzed the triacylglycerol lipase activity by detecting the free glycerol that was liberated from triglycerides. Glycerol is the final product of triglyceride hydrolysis, and it is generated after the degradation of monoglycerides. Interestingly, we found that *fgl1* had a severe defect in its ability to liberate free glycerol from triglycerides (Fig. 5A). The triacylglycerol lipase activities of *fglip1* and *fgl1* were restored in the complemented strains (Fig. S2). Since *fgl1* showed no visible defect in olive oil degradation on the rhodamine B plate (Fig. 3A), we hypothesized that *fgl1* might have a defect in the hydrolysis of diglycerides or monoglycerides and might have a preference for triglycerides.

We incubated 40 units (U) of culture supernatants of the wild-type, *fglip1*, and *fgl1* with triolein and analyzed the hydrolysis products, namely, oleic acids, monoolein, 1,2-diolein, 1,3-diolein, and triolein, via thin-layer chromatography (TLC) (Fig. 5B). We observed that the culture supernatants of all strains generated similar amounts of oleic acid but that monoolein was largely accumulated in *fgl1*, compared to the wild-type and *fglip1*. We subsequently analyzed the amount of free glycerol and found that the liberated glycerol was significantly lower in *fgl1* than in the wild-type (Fig. 5C). Our results support the proposal that Fgl1 might play a key role in the later stages of triolein hydrolysis. Since *fglip1* had no defect in the full hydrolysis of triolein, we estimated that although the degradation of triolein was slow, the later hydrolysis processes proceed quickly by Fgl1. We further analyzed the amount of oleic acid that was liberated from the triolein (Fig. 5D). Supporting our proposal, in *fglip1*, which was expected to have a low triolein degrading capacity, the production of oleic acid was slow at the beginning, whereas it was quickly generated in *fgl1*, which was expected to have a high preference for triolein. Taken together, these results indicate that FgLip1 has a substrate preference for triglycerides and that Fgl1 has a substrate preference for diglycerides or monoglycerides, which can act significantly when F. graminearum faces various lipid sources in the environment.

### Identification of transcription factors involved in lipase activity.

**FIG 6:**
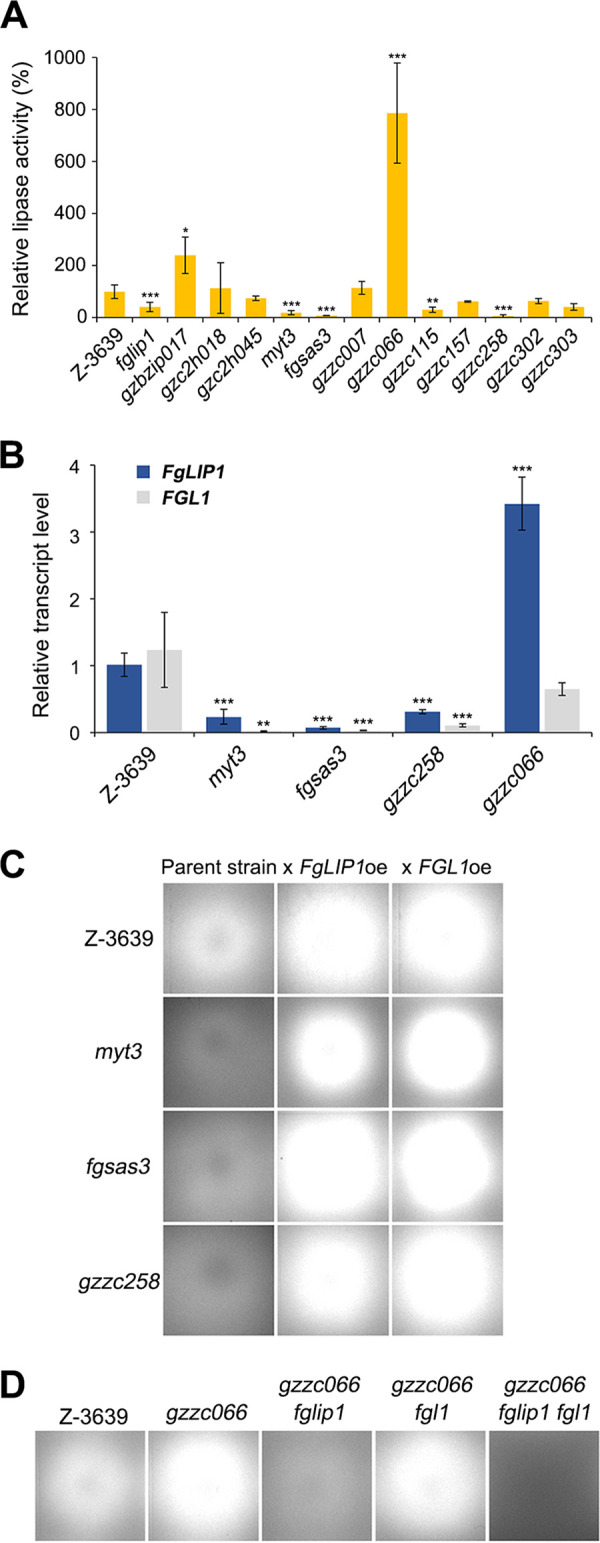
Characterization of transcription factors (TFs) involved in the lipase activity. (A) Lipase activity of 12 primarily selected TF mutants. The culture supernatant of each strain was harvested 12 h after inoculation in lipase-inducing medium. The lipase activity was measured spectrophotometrically, using pNPP as a substrate. Significant differences (*, *P* < 0.01; **, *P* < 0.005; ***, *P* < 0.001), compared to the wild-type, are indicated by asterisks. (B) Relative transcript levels of *FgLIP1* and *FGL1* in four TF mutants. Total RNA was extracted 12 h after inoculation in lipase-inducing medium. The relative transcript levels were analyzed via qRT-PCR. An asterisk indicates a significant difference (*, *P* < 0.01; **, *P* < 0.005; ***, *P* < 0.001) in the relative transcript level of each gene, compared to the wild-type. (C and D) Lipase activity of TF mutants carrying an *FgLIP1*, *FGL1* overexpression (C), or deletion (D). The strains were inoculated on MM containing 1% olive oil and 0.0005% rhodamine B. The pictures were taken 2 days after inoculation under UV light.

To identify the regulatory mechanisms of lipase activity in F. graminearum, we screened the lipase activity of 1,729 strains, corresponding to 657 transcription factor (TF) mutants (4) on MM containing olive oil and rhodamine B. 12 TF mutants exhibited altered fluorescence, compared to the wild-type (Fig. S3), and the lipase activities were measured using pNPP as a substrate (Fig. 6A). Among them, *myt3*, *fgsas3*, and *gzzc258*, which had the lowest lipase activity, and *gzzc066*, which had the highest lipase activity, were selected for further experiments. The putative Myb-like transcription factor Myt3 and the histone acetyltransferase FgSas3 have previously been characterized (47, 48). Gzzc258 was reported to affect the lipase activity in F. graminearum (49), and Gzzc066 encodes an ortholog of the transcriptional activator of gluconeogenesis Ert1 in yeast (50, 51).

We hypothesized that the selected TFs might regulate the transcription of *FgLIP1* or *FGL1* under lipase-inducing conditions. The relative transcript levels of *FgLIP1* and *FGL1* in the TF mutants were analyzed 12 h after induction via qRT-PCR (Fig. 6B)*. FgLIP1* and *FGL1* were markedly downregulated in *myt3*, *fgsas3*, and *gzzc258*, and the expression of *FgLIP1* was highly induced in *gzzc066*, in comparison with the wild-type.

We then overexpressed *FgLIP1* or *FGL1* in *myt3*, *fgsas3*, and *gzzc258*, and, conversely, we deleted these genes in *gzzc066* (Fig. 6C and D). The overexpression of *FgLIP1* or *FGL1* in *myt3*, *fgsas3*, and *gzzc258* rescued the defects in the lipase activity. The *gzzc066 fglip1* double mutant and the *gzzc066 fglip1 fgl1* triple mutant exhibited almost no fluorescence on the rhodamine B plate, whereas we could not observe a visible difference between *gzzc066* and the *gzzc066 fgl1* double mutant. The altered lipase activity of the selected TF mutants was restored in the complemented strains (Fig. S4), and the constitutive expression of *FgLIP1* and *FGL1* in the overexpressing strains was validated via qRT-PCR (Fig. S5A). These data indicate that several transcription factors, including *MYT3*, *FgSAS3*, *GzZC258*, and *GzZC066*, coregulates the lipase activity of F. graminearum by regulating *FgLIP1* and *FGL1*.

### Gzzc258 regulates lipase activity in a *FgLIP1*-dependent and *FGL1*-dependent manner.

**FIG 7:**
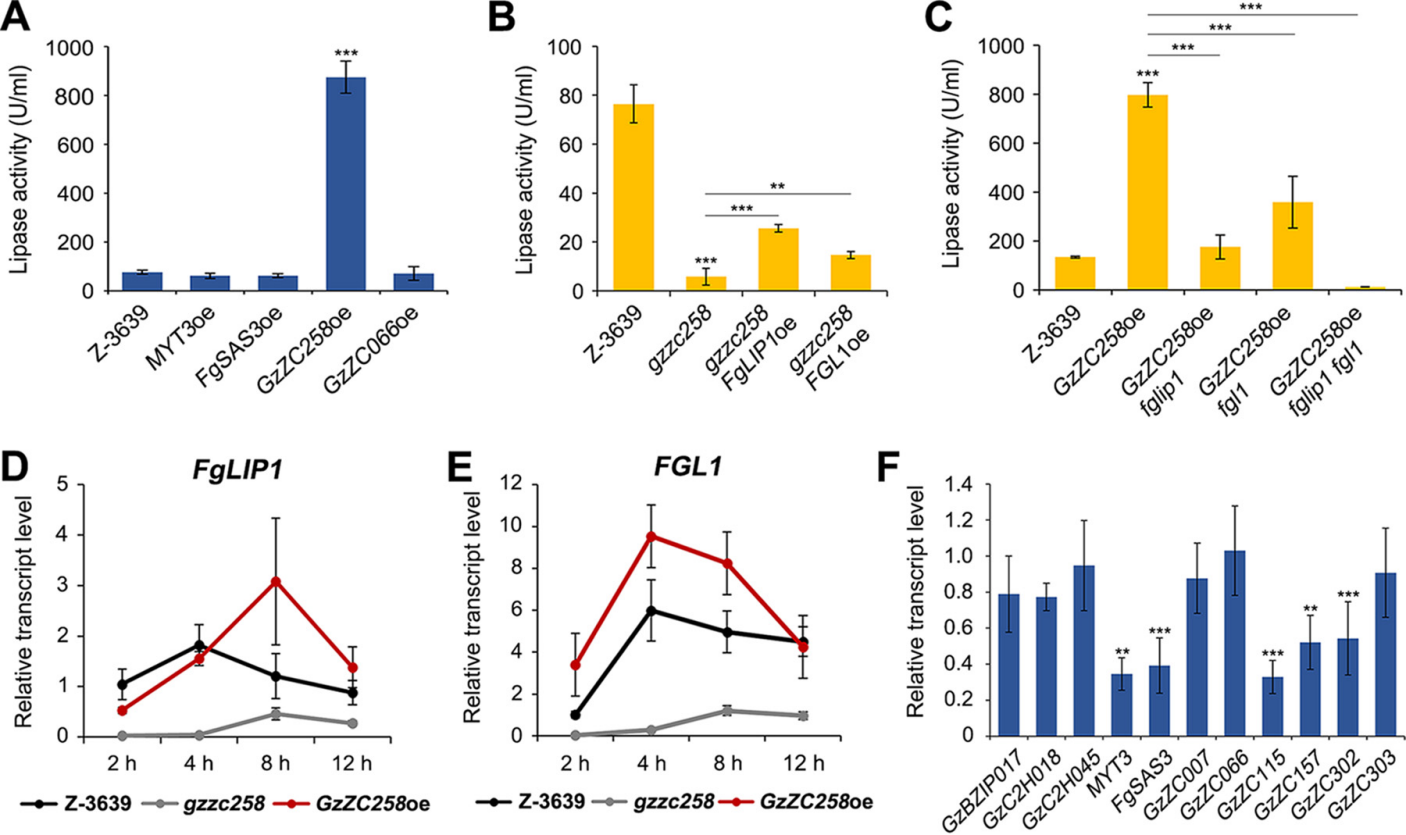
Gzzc258 as a key lipase regulator in F. graminearum. (A–C) Lipase activity of the overexpressing strains of four TF mutants (panel A), *gzzc258* carrying an *FgLIP1* or *FGL1* overexpression (panel B), and *GzZC258*oe carrying an *FgLIP1* or *FGL1* deletion (panel C). The strains were incubated for 12 h in lipase-inducing medium, and the culture supernatant was used for an analysis of the lipase activity, using pNPP as a substrate. An asterisk indicates a significant difference (**, *P* < 0.005; ***, *P* < 0.001), compared to the wild-type (panel A), gzzc258 (panel B), and *GzZC258*oe (panel C). (D and E) Transcript abundances of *FgLIP1* (panel D) and *FGL1* (panel E) in *GzZC258* deletion and overexpression mutants. Total RNA was extracted 2, 4, 8, and 12 h after inoculation in lipase-inducing medium, and the relative transcript levels were quantified via qRT-PCR. The relative transcript abundance of the indicated gene at 2 h after inoculation in Z-3639 was arbitrarily set to 1. (F) The relative transcript levels of the TF genes in *gzzc258.* Total RNA was extracted 2 h after cultivation in lipase-inducing medium, and the transcript abundances were quantified via qRT-PCR. An asterisk indicates a significant difference (**, *P* < 0.005; ***, *P* < 0.001), compared to the wild-type. The expressions of the indicated genes in Z-3639 were arbitrarily set to 1.

To identify a key lipase regulator among the selected TFs, we measured the lipase activity of the overexpressing strain of each TF gene 12 h after induction. The constitutive expression of each gene was validated via qRT-PCR (Fig. S5B). The lipase activity was induced to about 10-fold of that observed in the wild-type in the *GzZC258* overexpression mutant, whereas the overexpression of other genes made no measurable difference, indicating that Gzzc258 is a key lipase regulator in F. graminearum (Fig. 7A).

We subsequently focused on the regulatory role of Gzzc258 in the transcription of *FgLIP1* and *FGL1*. When *FgLIP1* or *FGL1* was overexpressed in *gzzc258*, the lipase activity was partially restored to about 4.5-fold and 2.5-fold, respectively, of that observed in *gzzc258* (Fig. 7B). When *FgLIP1* or *FGL1* was deleted in the *GzZC258* overexpression mutant, the lipase activity was largely decreased, compared to the overexpressing strain of *GzZC258* (Fig. 7C). In particular, when *FgLIP1* and *FGL1* were both deleted in the *GzZC258* overexpression mutant, the lipase activity was barely detectable.

We additionally analyzed the transcript levels of *FgLIP1* and *FGL1* in *gzzc258* and in the *GzZC258* overexpression mutant at 2, 4, 8, and 12 h after inoculation in the lipase-inducing medium (Fig. 7D and E). The transcript levels of *FgLIP1* and *FGL1* were barely detected in *gzzc258* and were recovered slightly after 8 h. In contrast, the *GzZC258* overexpressing strain had significantly higher transcript levels of *FgLIP1* and *FGL1* than did the wild-type. Taken together, the results demonstrate that Gzzc258 is a key lipase regulator that regulates lipase activity in a *FgLIP1*-dependent and *FGL1*-dependent manner.

**FIG 8:**
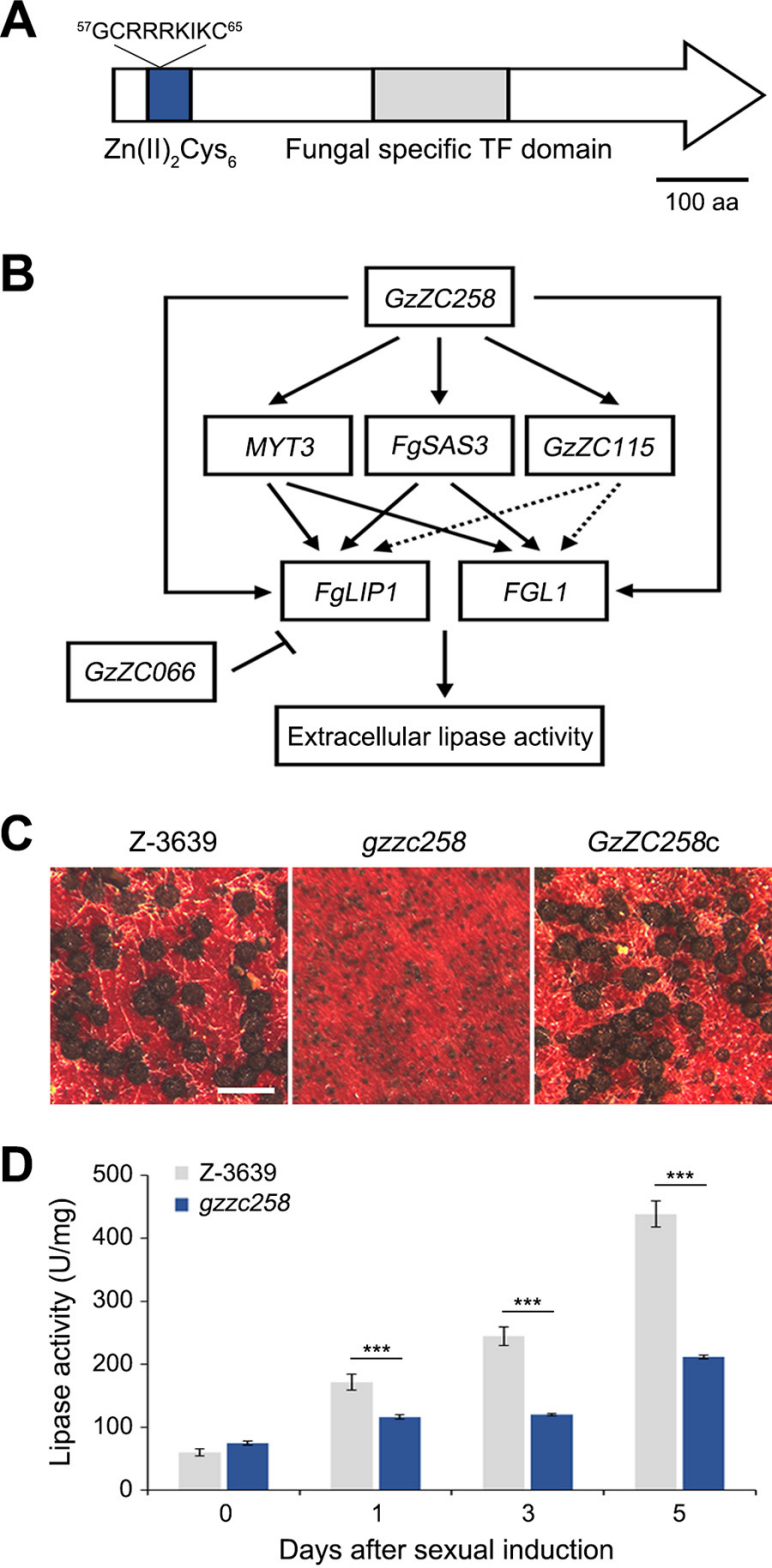
A schematic figure of the lipase regulatory mechanism in F. graminearum as well as the lipase activity in the sexual development stages. (A) Domain structure of Gzzc258. (B) Proposed genetic network of *FgLIP1*, *FGL1*, and TF genes. (C) Perithecium formation of *gzzc258* and the complemented strains. The photographs were taken 8 days after sexual induction on carrot agar. Scale bar = 500 μm. (D) Lipase activity of *gzzc258* in the sexual development stages. Whole-cell extracts were prepared from carrot agar cultures 0, 1, 3, and 5 days after sexual induction, and they were used for a lipase activity analysis, using pNPP as a substrate. An asterisk indicates a significant difference (***, *P* < 0.001) between the wild-type and *gzzc258* at each time period.

Gzzc258 encodes 889 amino acids and contains the Zn(II)_2_Cys_6_ DNA-binding domain (PF00172, IPR001138) and the fungal-specific transcription factor domain (PF04082, IPR007219) found in a number of fungal transcription factors, including XlnR, which regulates the extracellular cellulolytic and xylanolytic genes (52) (Fig. 8A). The nuclear localization signal, _57_GCRRRKIKC_65_, was predicted in Gzzc258 by NucPred (53) and suggested that Gzzc258 might act as a nuclear transcription factor in F. graminearum. We further analyzed the transcript levels of 12 primarily selected TF genes in *gzzc258.* We found that *MYT3*, *FgSAS3*, *GzZC115*, *GzZC157*, and *GzZC302* were markedly downregulated in *gzzc258* under lipase-inducing conditions (Fig. 7F). Our results indicate that Gzzc258 is a master lipase regulator in F. graminearum and that it affects the complex genetic networks that are involved in lipase activity. Based on the lipase activity of the TF mutants (Fig. 6A) and the altered transcript levels of the TF genes (Fig. 7F), we suggest a genetic network model, regarding the lipase activity in F. graminearum (Fig. 8B).

When the intracellular lipid droplets of *myt3*, *fgsas3*, *gzzc258*, and *gzzc066* were visualized via Nile red staining, we observed that the lipid droplet recovery was delayed in *gzzc258*, as in *fgl1* and *fglip1* (Fig. S6; Fig. 4). Interestingly, *myt3* and *fgsas3* were defective in degrading intracellular lipid droplets under starvation conditions, implying the involvement of Myt3 and FgSas3 in overall nutrient utilization.

### Lipase activity during the sexual development stages.

F. graminearum accumulates the lipid bodies in the early perithecial formation, and lipid oxidation processes occur in the later stages of sexual development (34, 35). We observed the sexual reproduction of the selected TF mutants to find the relationship between lipase activity and sexual development. *myt3* and *fgsas3* failed to produce any perithecia, and *gzzc258* only developed immature and tiny perithecial structures after sexual induction. *gzzc066* was not able to discharge ascospores at all. (Fig. 8C; Fig. S7). We subsequently analyzed the lipase activities of *gzzc258* and the wild-type at 0, 1, 3, and 5 days after sexual induction. (Fig. 8D). The lipase activity of the wild-type rapidly increased after sexual induction and finally reached about 7-fold of that of the vegetative stages. *gzzc258* showed delayed enzymatic induction, and the lipase activity only increased to about 3-fold of that of the vegetative stages. Our results suggest that lipase activity is induced in the sexual development stages and is required for perithecial maturation.

## DISCUSSION

We identified a total of 86 putative lipase-encoding genes with conserved lipase or esterase domains and analyzed the phenotypic changes of 86 putative lipase deletion mutants in various developmental processes. Seven mutants, namely, *fg08139*, *fg05297*, *fg00208*, *fgpld1*, *tri8*, *fg07372*, and *fg12678*, showed defects in the analyzed phenotypes (Fig. 1 and 2; Table S1), especially in vegetative growth and sexual development. With the exception of *tri8*, which only showed a defect in DON production (54), all genes but *Fg12678* belonged to phospholipase families. These results indicate that most lipases play overlapping roles in developmental processes. Therefore, a single gene disruption does not lead to significant phenotypic defects. Research on intracellular lipases is commonly conducted via the generation of double or triple mutants in yeast (55, 56). We found that three ortholog genes of Saccharomyces cerevisiae Tgl3, which is a main enzyme that is responsible for intracellular lipase activity, exists in F. graminearum with a significant match (expect <1E^−30^) (57). Based on this, we expect that a single lipase gene would play a dispensable role in F. graminearum, but a double or triple mutant would lead to defects in several biological processes.

None of the mutants, including *fgl1*, showed defects in virulence on wheat spikelets in our laboratory conditions, except for *fgpld1*. Previous studies have also demonstrated the dispensable roles of fungal lipases in virulence in M. oryzae, F. oxysporum, and *B. cinerea* (31–33). We expect either that the functional redundancy of the lipase genes exists in the virulence of F. graminearum or that the majority of lipase genes contribute to nutritional uptake from the plant tissue, not to fungal virulence.

FgLip1 and Fgl1 are previously reported extracellular lipases in F. graminearum that are highly induced during the infection stages and in the presence of lipid sources, such as olive oil and wheat germ oil (24, 29). FgLip1 and Fgl1 both harbor the signal peptides in their N termini of 15 amino acid residues and 20 amino acid residues, respectively, suggesting that they function as secretory lipases. We investigated the extracellular lipase activities of the putative lipase mutants (Fig. 3). Only *fglip1* had defects in the decomposition of the olive oil on the plate, indicating that the majority of the extracellular triglyceride lipase activity of F. graminearum depends on FgLip1. When we quantified the lipase activities of the strains, using pNPP as a substrate, a single deletion of *FgLIP1* or *FGL1* showed significant defects, compared with the wild-type. We further demonstrated in Fig. 4 that these lipases hydrolyze extracellular olive oil, which allows the cells to utilize the extracellular lipid sources. When olive oil was supplemented as a sole carbon source, the wild-type initially used the internal lipids as carbon sources, but the intracellular lipid droplets began to accumulate again as extracellular lipases decomposed the external olive oil. However, when *FgLIP1* or *FGL1* was deleted, the mutant lacked the ability to decompose external olive oil. So, the cells continued to use internal lipids, resulting in defects in the recovery of internal lipid droplets. This result allowed us to visually demonstrate how F. graminearum uses lipids inside and outside the cell when either carbon sources are scarce or external lipid sources are present. Despite several extracellular lipases, other than FgLip1 and Fgl1, having been characterized in F. graminearum (58), we conclude that FgLip1 and Fgl1 might constitute most of the lipase activity in F. graminearum, as *fglip1 fgl1* had no pNPP hydrolysis activity in our study. However, other results can be obtained if the lipases are induced with other lipid sources or if the activity is analyzed for other substrates, such as pNP-butyrate.

*fgl1* had a severe defect in generating glycerol from triglycerides, which means a defect in the full hydrolysis of triglycerides (Fig. 5A). Based on our results, we could hypothesize that (i) FgLip1, which is expected to account for most of the activity in *fgl1*, would primarily act on the hydrolysis of triglycerides and have lower activity on the later substrates and that (ii) Fgl1 would have a higher preference for the later substrates than did FgLip1. Our TLC analysis shows that monoglycerides are accumulated in *fgl1*, which supports our proposal (Fig. 5B). To analyze the detailed enzymatic characteristics of the lipases, further research is needed, specifically in reacting the purified enzymes with various substrates.

Although we demonstrated that FgLip1 and Fgl1 are required for the extracellular lipase activity in F. graminearum, we could not find any defects in the assessed phenotypes. Plant-pathogenic fungi encounter various environments in their life cycles. Previous studies suggest that plant-pathogenic fungi face nutrient deprivation during their proliferation in plant tissues (59, 60). After the infection of the wheat florets, F. graminearum colonizes the wheat stems and produces the specified hyphal coils, termed perithecium initials, for overwintering (61). For successful colonization, nutrient uptake from the plant tissue via the secretion of various degrading enzymes is required (62–64). A previous study revealed the induced expression of lipases, including FgLip1 and Fgl1, during wheat colonization (34). So, we expect that extracellular lipases in F. graminearum might contribute to fungal survival outside laboratory conditions.

We identified three transcription factors, namely, Myt3, Gzzc258, and Gzzc066, and one histone acetyltransferase, namely, gSas3, that were involved in the lipase activity in F. graminearum. Previously, Ctf1α/β in Fusarium species, which are homologous to FarA and FarB in A. nidulans, have been characterized as a cutinase and lipase regulator in several fungi (33, 49, 65–68). Among the 12 primarily selected TFs, Gzzc115 and Gzzc258 had sequence similarities of up to 84% and 19% with the Ctf1α of Fusarium solani, respectively. We found that *myt3*, *fgsas3*, *gzzc258*, and *gzzc066* showed altered transcript levels of *FgLIP1* and *FGL1*, and the overexpression or deletion of *FgLIP1* or *FGL1* directly recovered their lipase activities (Fig. 6B and C). These results suggest that the altered lipase activities of the TF mutants were due not to the defects in secretion or posttranslational modification but to the transcriptional defects of *FgLIP1* and *FGL1*. Among the selected TFs, we designated Gzzc258 as a key lipase regulator, as the overexpression of *GzZC258* led to the enhanced lipase activity and the increased transcription of *FgLIP1* and *FGL1* (Fig. 7). We found that multiple TF genes that are involved in lipase activity, including *MYT3* and *FgSAS3*, were downregulated in *gzzc258* under the lipase-inducing conditions, suggesting a complex genetic network of the lipase regulatory genes.

In this study, we demonstrated that F. graminearum greatly increases its lipase activity during its sexual developmental stages, whereas *gzzc258* only produced immature perithecial initials with lower induction of the lipase activity (Fig. 8B and C). Since *fglip1*, *fgl1*, and even *fglip1 fgl1* successfully developed mature perithecia (data not shown), we conclude that intracellular, rather than extracellular, lipases might act for the cellular lipid accumulation and degradation during sexual reproduction stages.

In conclusion, we constructed genome-wide deletion mutants of putative lipases in F. graminearum and provided a phenotypic database of the mutants. Our study shows that F. graminearum possesses abundant lipases that work for cellular growth and differentiation. We identified several TFs that coregulate the expression of the extracellular lipases FgLip1 and Fgl1, and we designated Gzzc258 as a key lipase regulator. We revealed the relationship between lipase activity and sexual development in F. graminearum, thereby suggesting the potential roles for lipases in various cellular mechanisms. Our study provides extensive genetic sources regarding fungal lipases and reveals the complex mechanisms that regulate extracellular lipase activity in plant-pathogenic fungi.

## MATERIALS AND METHODS

### Strains and culture conditions.

The F. graminearum wild-type strain Z-3639 (69) was used as the parental strain for the transformation experiments. All strains were stored as mycelial suspensions in a 20% glycerol solution at −80°C. The TF deletion mutants as well as the complementation and overexpressing strains of *MYT3* that were used in this study have been constructed previously (4, 47). The culture media were prepared following the Fusarium laboratory manual (1). Conidial production was induced in carboxymethyl cellulose (CMC) medium or on yeast malt agar (YMA) (70, 71). For the lipase activity assay, MM supplemented with 1% olive oil as a sole carbon source was emulsified using a Sonics VCX750 ultrasonic processor (Sonics & Materials, Newtown, CT, USA). The growth temperature for fungal strains was set at 25°C.

### Nucleic acid manipulations, Southern blotting, and PCR.

The genomic DNA was extracted as previously described (1). Total RNA was extracted from mycelia ground in liquid nitrogen using an Easy-Spin Total RNA Extraction Kit (Intron Biotech, Seongnam, Republic of Korea). Standard protocols were used for the restriction endonuclease digestion and agarose gel electrophoresis (72). Southern blot hybridization was performed using a North2South Biotin Random Prime Labeling Kit and a North2South Chemiluminescent Hybridization and Detection Kit (Thermo Scientific, USA) or by following standard techniques (72). The PCR primers (Table S2) were synthesized by an oligonucleotide synthesis facility (Bionics, Seoul, Republic of Korea).

### Genetic manipulations and fungal transformations.

The double-joint (DJ) PCR method (73) was used to generate the fusion PCR products that were required for the gene deletion, complementation, and overexpression. The fungal transformation was performed as previously described (74).

To construct the deletion mutants, the 5′ and 3′ flanking regions of the target genes were amplified from the genomic DNA, and the Geneticin resistance gene cassette (*GEN*) was amplified from pII99 (75). Three fragments were fused via the DJ PCR method, and the final constructs were amplified using the nested primers. The resulting amplicons were transformed into the fungal wild-type protoplasts, and the mutants were confirmed via Southern hybridization with a *GEN* probe, a flanking region probe, or a probe located in the ORF region.

For the complementation of the *gzzc258* deletion mutant, the 5′ flanking region, including the open reading frame (ORF) with its native promoter, and the 3′ flanking region were amplified from the genomic DNA of the wild-type strain. The *GFP-HYG* construct carrying the green fluorescent protein (*GFP*) gene and the hygromycin resistance gene cassette (*HYG*) was amplified from pIGPAPA (76). The three amplicons were fused, and the final constructs were obtained using nested primers. The resulting amplicons were introduced into the *gzzc258* deletion mutant as previously described (77), and the mutants were confirmed via Southern blotting (Fig. S8).

To generate the *GzZC258* overexpression mutant, the 5′ flanking region and the ORF of GzZC258 were amplified from the genomic DNA, and the *GEN-P _ef1α_* construct carrying *GEN* and the elongation factor 1α promoter (*P_EF1α_*) were amplified from pSKGEN (36). The resulting products were fused, and the final PCR constructs were amplified with nested primers. The fusion constructs were used to transform fungal wild-type protoplasts. Transformants constructed via DJ PCR and homologous recombination methods were all confirmed via Southern blotting (Fig. S8).

To complement the *fglip1* deletion mutant, the ORF of *FgLIP1* and its own promoter were amplified and fused with *HYG* that was amplified from pIGPAPA. The fusion construct was randomly integrated into the *fglip1* deletion mutant.

For the complementation of the *fgsas3* deletion mutant, the *FgSAS3*-*GFP* fusion construct was generated via the yeast gap repair approach (78). The ORF of *FgSAS3* and its native promoter were amplified from the genomic DNA of the wild-type strain. The resulting construct and XhoI-digested pDL2 were cotransformed into the yeast strain PJ69-4A (79) using a Alkali-Cation Yeast Transformation Kit (MP Bio, Seoul, Republic of Korea). The *FgSAS3*-*GFP* fusion vector that was obtained from the yeast transformants was transformed into Escherichia coli DH10B. After verification via sequencing, the plasmid DNA was extracted with a DNA-spin Plasmid DNA Purification Kit (Intron Biotech, Seongnam, Republic of Korea) and used to transform the *fgsas3* deletion mutant. The complementation of the *gzzc066* and *fgl1* deletion mutants was performed via the same strategy.

To overexpress *FgSAS3*, the ORF was amplified and cotransformed with XhoI-digested pDL2 into the yeast strain PJ69-4A, as described above. The *RP27*-*FgSAS3-GFP* fusion vector carrying the M. oryzae ribosomal protein 27 promoter (80) was obtained from the yeast transformants. The subsequent process was carried out in the same way. The verified vectors were transformed into the deletion mutant or the wild-type strain. The overexpression mutant of *GzZC066* was generated using the same strategy, and, for *FGL1* and *FgLIP1*, the overexpressing strains were constructed without the *GFP* construct.

To overexpress *FGL1* and *FgLIP1* in the TF deletion mutants, *FGL1* and *FgLIP1* overexpression vectors were transformed into the TF deletion mutants, respectively. All of the overexpressing strains were confirmed via qRT-PCR.

### Vegetative growth, conidiation, and sexual development.

Radial growth and colony morphology were assayed on CM and MM 4 to 5 days after inoculation. For the conidiation assays, a fresh mycelial plug from CM was inoculated in 5 mL of CMC for 5 days on a rotary shaker (200 rpm). The number of conidia was measured with a hemacytometer.

For sexual development, fungal strains were grown on carrot agar for 5 days, and aerial mycelia were removed with 0.4 mL of a sterile 2.5% Tween 60 solution (1). The plates were then incubated under near-UV light (wavelength: 365 nm; Sankyo Denki, Tokyo, Japan). The number of perithecia, maturation, ascospore morphology, and ascospore discharge were observed after 7 to 9 days.

For outcrosses, the female strain was fertilized with 1 mL of a conidial suspension from the male strain 5 days after inoculation on carrot agar.

### Stress response.

The stress responses were evaluated by assaying vegetative growth on CM supplemented with various stress agents: oxidative stress (6 mM hydrogen peroxide and 40 μM menadione), osmotic stress (1 M NaCl, 1 M KCl, 1.5 M sorbitol, 4 mM FeSO_4_), pH stress (pH 4 and pH 11), cell wall stress (60 mg/L Congo red and 100 mg/L sodium dodecyl sulfate), DNA synthesis inhibition (7 mg/L iprodione), the inhibition of mitogen activated protein kinase (0.02 mg/L fludioxonil), the inhibition of meiosis and intracellular transportation (0.65 mg/L benomyl), and azole fungicide (0.025 mg/L tebuconazole) (4).

### Virulence test and mycotoxin analysis.

The point inoculation method was performed to assay fungal virulence as previously described (74). Conidia were harvested from CMC cultures, and 10 μL of each suspension (10^5^ conidia/mL) was injected into the center spikelet of a wheat head (cultivar: Eunpamil). The inoculated wheat plants were incubated in a humid chamber for 3 days and grown in a greenhouse for an additional 18 days. The number of diseased spikelets was measured 21 days after inoculation.

The DON and ZEA extraction was performed as previously described (81). A fresh mycelial plug was inoculated on 1.5 g of rice substrate for 3 weeks. The rice culture was harvested, ground, and mixed vigorously with 6 mL of 84% acetonitrile for 30 min. After phase separation, the upper phase was filtered through a 0.45 μm syringe filter. Reverse-phase HPLC on a Prominence HPLC system (Shimadzu, Japan) with a C18 column was used for the analysis, with a simple modification of the previous methods (82, 83). For ZEA detection, the mobile phase was 70% aqueous methanol, and the flow rate was 1 mL min^−1^. For DON detection, the mobile phase was 10% aqueous acetonitrile (ACN), and the flow rate was 1 mL min^−1^. A gradient elution program was applied as follows: after 10% ACN was maintained for 11 min, it was linearly increased to 30% ACN at 12 min. It was then linearly decreased from 30% ACN at 12 min to 10% ACN at 18 min. Subsequently, 10% ACN was held for 17 min for the reequilibration of the column before the injection of the next sample, giving a total run time of 35 min. The diode-array detection was applied (ZEA and DON were detected at a wavelength of 235 nm).

### Lipase activity assay.

Lipase activity was detected using rhodamine B (84). MM supplemented with 1% olive oil (vol/vol) as a sole carbon source was emulsified, and rhodamine B was added to a final concentration of 0.0005% (wt/vol). The fresh mycelial plug was inoculated on the rhodamine B plates, and the fluorescence was observed 2 days after inoculation under UV light. For screening the lipase activity of the TF mutants, vitamin stock solution (1) was added to the medium so that the fluorescence could be clearly observed.

For the quantitative lipase activity assay, a para-nitrophenyl palmitate (pNPP) assay was performed (45). The mycelia were cultured in lipase-inducing conditions, and a portion of the culture supernatant was mixed with 200 μL of the reaction buffer containing 0.79 mM pNPP, 0.1% (vol/vol) Triton X-100, 0.1% (wt/vol) gum arabic, and 50 mM tris-Cl, pH 7.5. The plate was incubated at 37°C. The amount of released para-nitrophenol (pNP) was measured spectrophotometrically at 410 nm. One unit (U) of lipase activity was defined as 1 μmol of pNP released per minute. For mycelia harvested from carrot agar cultures, the tissue was ground in liquid nitrogen, and the whole-cell extract was obtained with extraction buffer (50 mM Tris-Cl, pH 8.0, 150 mM NaCl, 5% glycerol, 1% NP-4, 1 mM EDTA, 1 mM PMSF, 2 μg/mL Leupeptin, 2 μg/mL pepstatin A, 20 μg/mL aprotinin). After centrifugation at 13,000 rpm for 20 min, the supernatant was used for the lipase activity assay as described above. The total protein concentration was determined using the Bradford method (85).

Triacylglycerol lipase activity was measured with a Lipase Assay Kit (MAK046, Sigma). One unit (U) of lipase was defined as the amount of enzyme needed to liberate 1 μmol of glycerol from triglycerides per minute.

To analyze the lipolysis product, 1 mL of reaction mixture containing 10 μL triolein, 50 mM tris-Cl buffer, pH 7.5, and 40 U of culture supernatant, as estimated via pNPP assay, was incubated at 37°C for 20 h on a rotary shaker (200 rpm). Total lipids were extracted with chloroform/methanol (2:1, vol/vol) and applied to silica gel 60 TLC plates (Merck, Darmstadt, Germany). The plates were developed with a solvent mixture of hexane/diethyl ether/acetic acid (70:30:1, vol/vol/vol). The spots were visualized using para-anisaldehyde stain and compared with reference standards from Sigma (Supelco mono-, di- and tri-glycerol mix, 1787-1AMP; oleic acid, O1383). The amount of free glycerol in the reaction mixture was measured using a Free Glycerol Assay Kit (ab65537, Abcam).

To analyze the amount of oleic acids that was liberated during the hydrolysis of triolein, the culture supernatant with 50 U of lipase was mixed with 40 mM triolein dissolved in isooctane in 50 mM tris-Cl buffer, pH 7.5. The reaction was initiated by adding 40 mM triolein dissolved in isooctane to form an aqueous/isooctane biphasic system. The reaction mixture was incubated at 37°C for 0, 4, 12, 20, 30, 35, 40, and 48 h with vigorous mixing. Total lipids were extracted with acetonitrile:acetone (90:10, vol/vol), and a portion of the lipid extracts was used to analyze the number of oleic species. The Chiralpak IA column (Daicel Chemical Ind., Osaka, Japan) was loaded on a Waters Alliance e2695 separation module-HPLC (Waters, Milford, MA, USA) and an Alltech ELSD 2000 instrument (Alltech Co., Deerfield, MA, USA) system, following the previously described method (86).

### Visualization of lipids and microscopic observation.

Conidial suspensions were inoculated in CM at 2 × 10^5^ conidia per milliliter, and mycelia were harvested 24 h after incubation on a rotary shaker (200 rpm). The mycelia were recultured in MM, MM without a carbon source, and MM supplemented with olive oil as the sole carbon source for 6, 12, and 24 h. The harvested mycelia were stained with Nile red solution (0.01 mg/mL in acetone) for 5 min (87). Microscopic observation was performed with a Leica DM6 B microscope (Leica Microsystems, Wetzlar, Germany) that was equipped with a Leica DMC6200 camera using the fluorescent filter Y3 (part no. 11504169). The perithecia were imaged 8 days after sexual induction using a Zeiss SteREO Lumar.V12 microscope (Carl Zeiss, Germany).

### qRT-PCR.

Total RNA was prepared using an Easy-Spin Total RNA Extraction Kit (Intron Biotech, Seongnam, Republic of Korea). First strand cDNA was synthesized from total RNA using SuperScript III reverse transcriptase (Invitrogen, Carlsbad, CA, USA). qRT-PCR was carried out using SYBR green Supermix (Bio-Rad, Hercules, CA, USA) and with a CFX real-time PCR system (Bio-Rad), using the corresponding primers (Table S2). The endogenous housekeeping gene ubiquitin C-terminal hydrolase (*UBH*) was used as a reference gene. The PCR was repeated three times with two biological replicates. The relative transcript levels of the target genes were calculated as previously described (87).

Conidial suspensions were inoculated in CM at 2 × 10^5^ conidia per milliliter. Mycelia were harvested 24 h after incubation on a rotary shaker (200 rpm) and washed twice with distilled water. The mycelia were recultured in MM supplemented with olive oil as the sole carbon source. Total RNA was isolated at the indicated time points, and the relative transcript levels were analyzed via qRT-PCR. For the overexpressing strains, conidial suspensions were inoculated in CM at 5 × 10^5^ conidia per milliliter, and total RNA was extracted after 24 h of cultivation. The constitutive expression of each gene was confirmed via qRT-PCR.
