# Supplementary material for: Genetic and Transcriptional Regulatory Mechanisms of Lipase Activity in the Plant Pathogenic Fungus Fusarium graminearum
Source: Microbiol Spectr. 2023 Apr 24;11(3):e05285-22. doi: 10.1128/spectrum.05285-22 (PMC10269793; doi:10.1128/spectrum.05285-22)
Supplement: Supplemental file 1 — Fig. S1 to S8. Download spectrum.05285-22-s0001.pdf, PDF file, 1.6 MB [file spectrum.05285-22-s0001.pdf]

Supplemental material

Genetic and transcriptional regulatory mechanisms of lipase activity in the plant pathogenic fungus *Fusarium graminearum*

Sieun Kim[1], Juno Lee[1], Jiyeun Park[1], Soyoung Choi[1], Duc-Cuong Bui[2], Jung-Eun Kim[3], Jiyoung Shin[4], Hun Kim[5], Gyung Ja Choi[5], Yin-Won Lee[1], Pahn-Shick Chang[1,6,7,8], and Hokyoung Son[1,6*]

[1]Department of Agricultural Biotechnology, Seoul National University, Seoul, Republic of Korea

[2]Department of Pathology, University of Texas Medical Branch, Galveston, Texas, USA

[3]Research Institute of Climate Change and Agriculture, National Institute of Horticultural and Herbal Science, Jeju, Republic of Korea

[4]Division of Bioresources Bank, Honam National Institute of Biological Resources, Mokpo, Republic of Korea

[5]Center for Eco-friendly New Materials, Korea Research Institute of Chemical Technology, Daejeon, Republic of Korea

[6]Research Institute of Agriculture and Life Sciences, Seoul National University, Seoul, Republic of Korea

[7]Center for Food and Bioconvergence, Seoul National University, Seoul, Republic of Korea

[8]Center for Agricultural Microorganism and Enzyme, Seoul National University, Seoul, Republic of Korea

Correspondence

Hokyoung Son, Department of Agricultural Biotechnology, Seoul National University, Seoul, Republic of Korea.

Email: hogongi7@snu.ac.kr

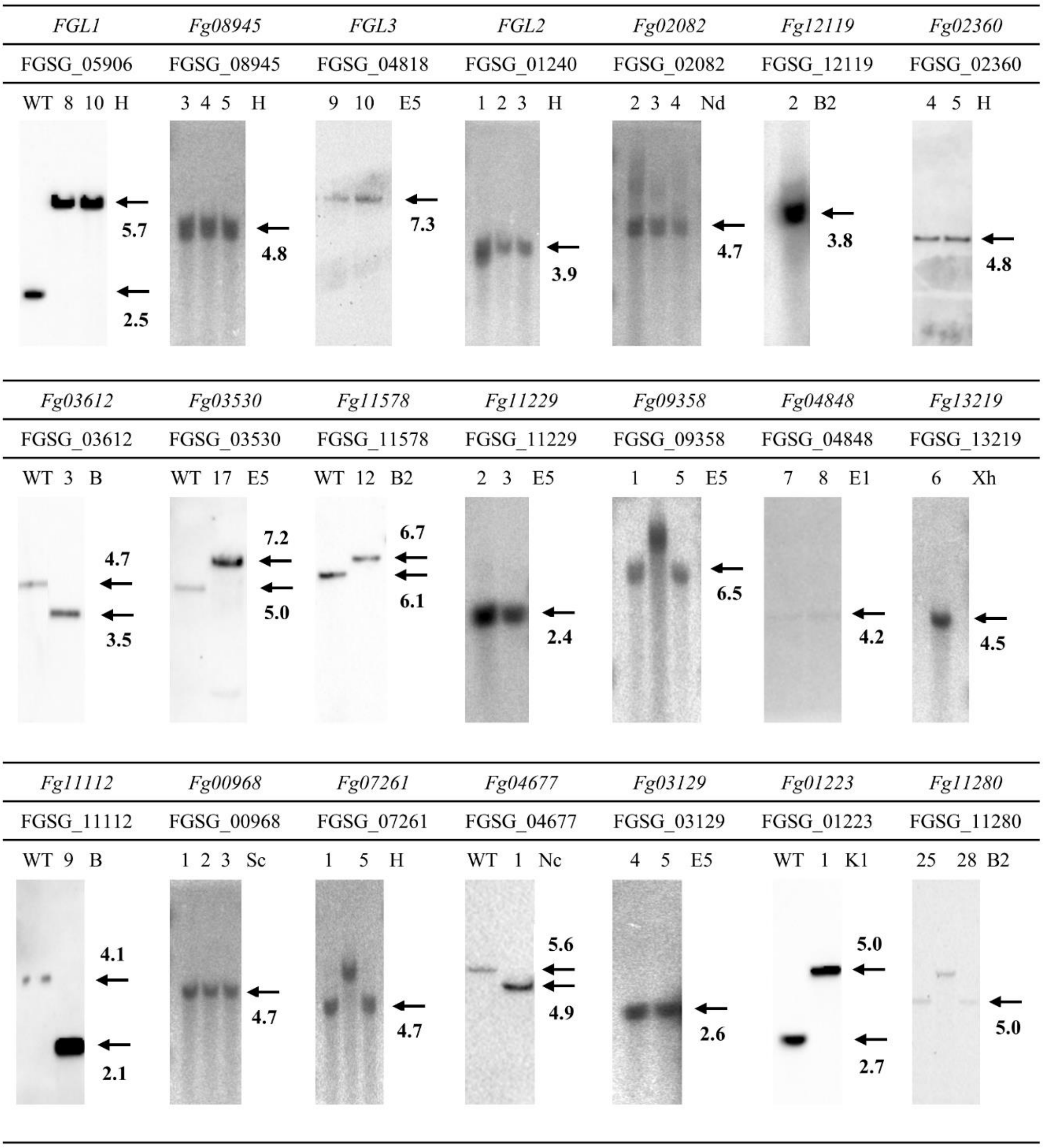

Figure S1. Confirmation of lipase deletion mutants by Southern blot analysis. The restriction enzymes used for each blot and the size of the DNA standards (kb) are indicated on the right of each blot. H, HindIII; E1, EcoRI; E5, EcoRV; B, BamHI; B1, BglI; B2, BglII; S, SalI; Sc, SacI; Nd, NdeI; Xh, XhoI; Xb, XbaI; P1, PstI; C1, ClaI; K1, KpnI; A1, AccI; Ss, SspI; WT, *F. graminearum* wild-type strain Z-3639.

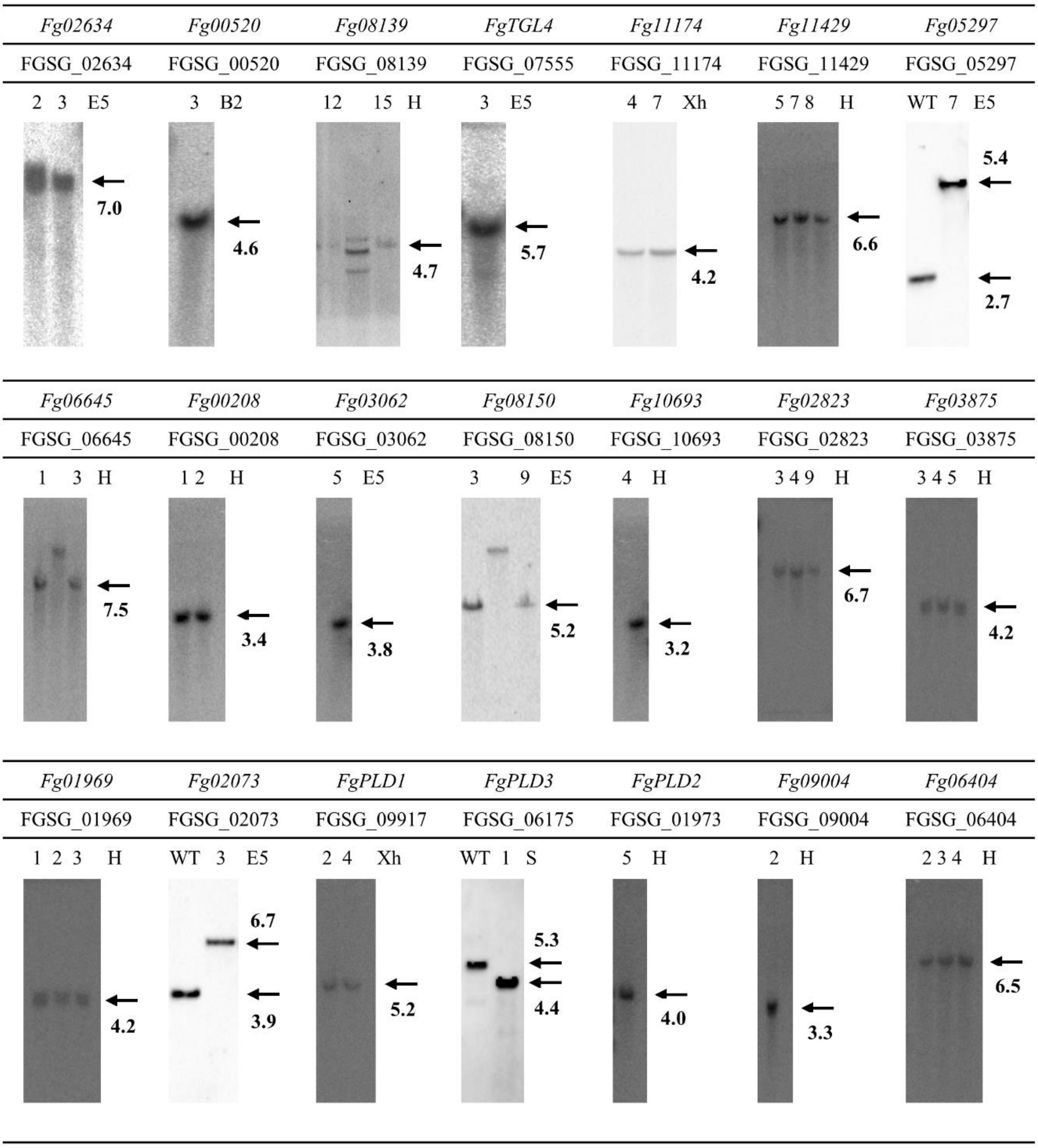

Fg02634
FGSG_02634
2 3 E5
7.0
Fg00520
FGSG_00520
3 B2
4.6
Fg08139
FGSG_08139
12 15 H
4.7
FgTGL4
FGSG_07555
3 E5
5.7
Fg11174
FGSG_11174
4 7 Xh
4.2
Fg11429
FGSG_11429
5 7 8 H
6.6
Fg05297
FGSG_05297
WT 7 E5
5.4
2.7
Fg06645
FGSG_06645
1 3 H
7.5
Fg00208
FGSG_00208
1 2 H
3.4
Fg03062
FGSG_03062
5 E5
3.8
Fg08150
FGSG_08150
3 9 E5
5.2
Fg10693
FGSG_10693
4 H
3.2
Fg02823
FGSG_02823
3 4 9 H
6.7
Fg03875
FGSG_03875
3 4 5 H
4.2
Fg01969
FGSG_01969
1 2 3 H
4.2
Fg02073
FGSG_02073
WT 3 E5
6.7
3.9
FgPLD1
FGSG_09917
2 4 Xh
5.2
FgPLD3
FGSG_06175
WT 1 S
5.3
4.4
FgPLD2
FGSG_01973
5 H
4.0
Fg09004
FGSG_09004
2 H
3.3
Fg06404
FGSG_06404
2 3 4 H
6.5

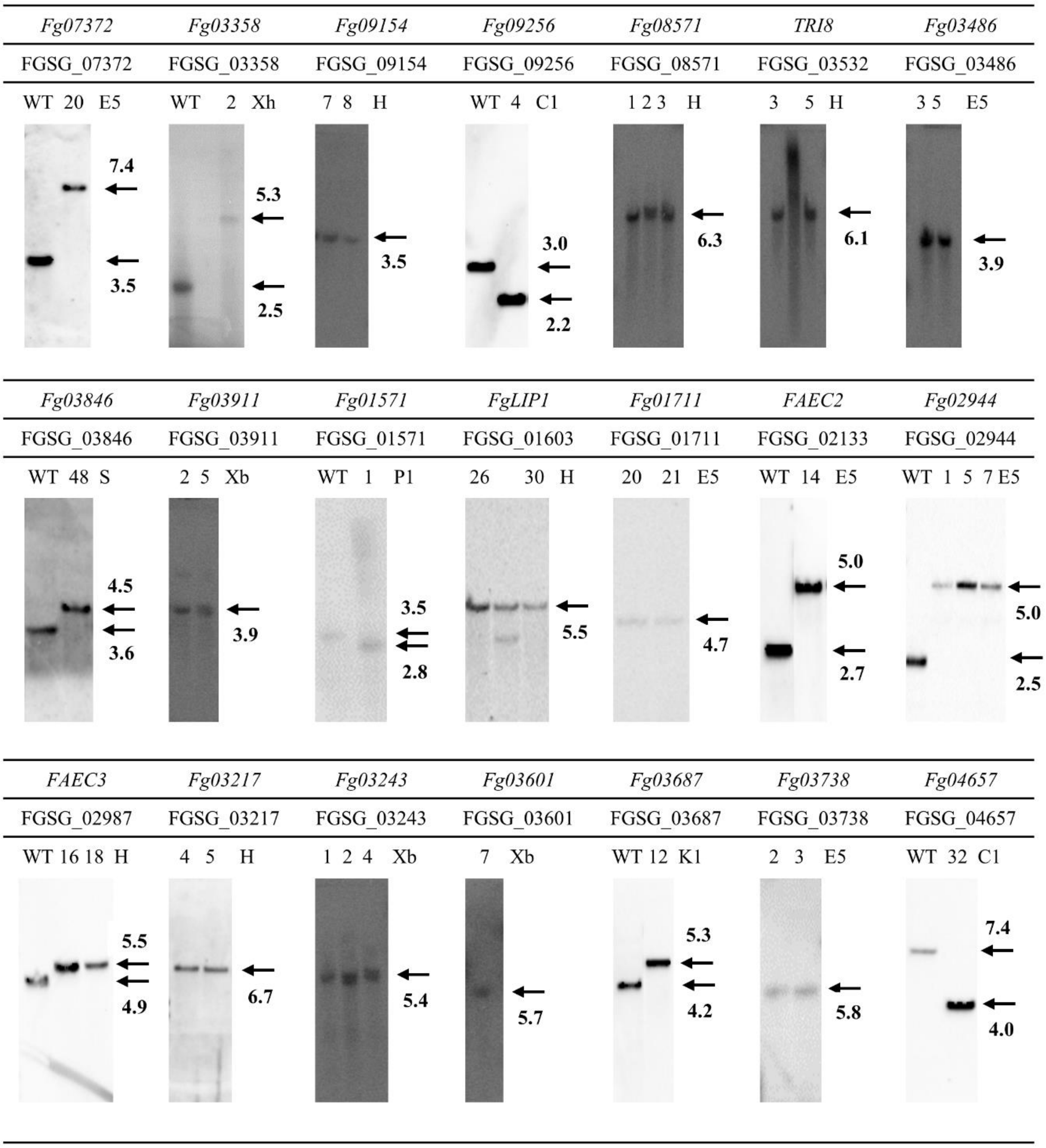
Fg07372 Fg03358 Fg09154 Fg09256 Fg08571 TRI8 Fg03486
FGSG_07372 FGSG_03358 FGSG_09154 FGSG_09256 FGSG_08571 FGSG_03532 FGSG_03486
WT 20 E5
7.4
3.5
WT 2 Xh
5.3
2.5
7 8 H
3.5
WT 4 C1
3.0
2.2
1 2 3 H
6.3
3 5 H
6.1
3 5 E5
3.9
Fg03846 Fg03911 Fg01571 FgLIP1 Fg01711 FAEC2 Fg02944
FGSG_03846 FGSG_03911 FGSG_01571 FGSG_01603 FGSG_01711 FGSG_02133 FGSG_02944
WT 48 S
4.5
3.6
2 5 Xb
3.9
WT 1 P1
3.5
2.8
26 30 H
5.5
20 21 E5
4.7
WT 14 E5
5.0
2.7
WT 1 5 7 E5
5.0
2.5
FAEC3 Fg03217 Fg03243 Fg03601 Fg03687 Fg03738 Fg04657
FGSG_02987 FGSG_03217 FGSG_03243 FGSG_03601 FGSG_03687 FGSG_03738 FGSG_04657
WT 16 18 H
5.5
4.9
4 5 H
6.7
1 2 4 Xb
5.4
7 Xb
5.7
WT 12 K1
5.3
4.2
2 3 E5
5.8
WT 32 C1
7.4
4.0

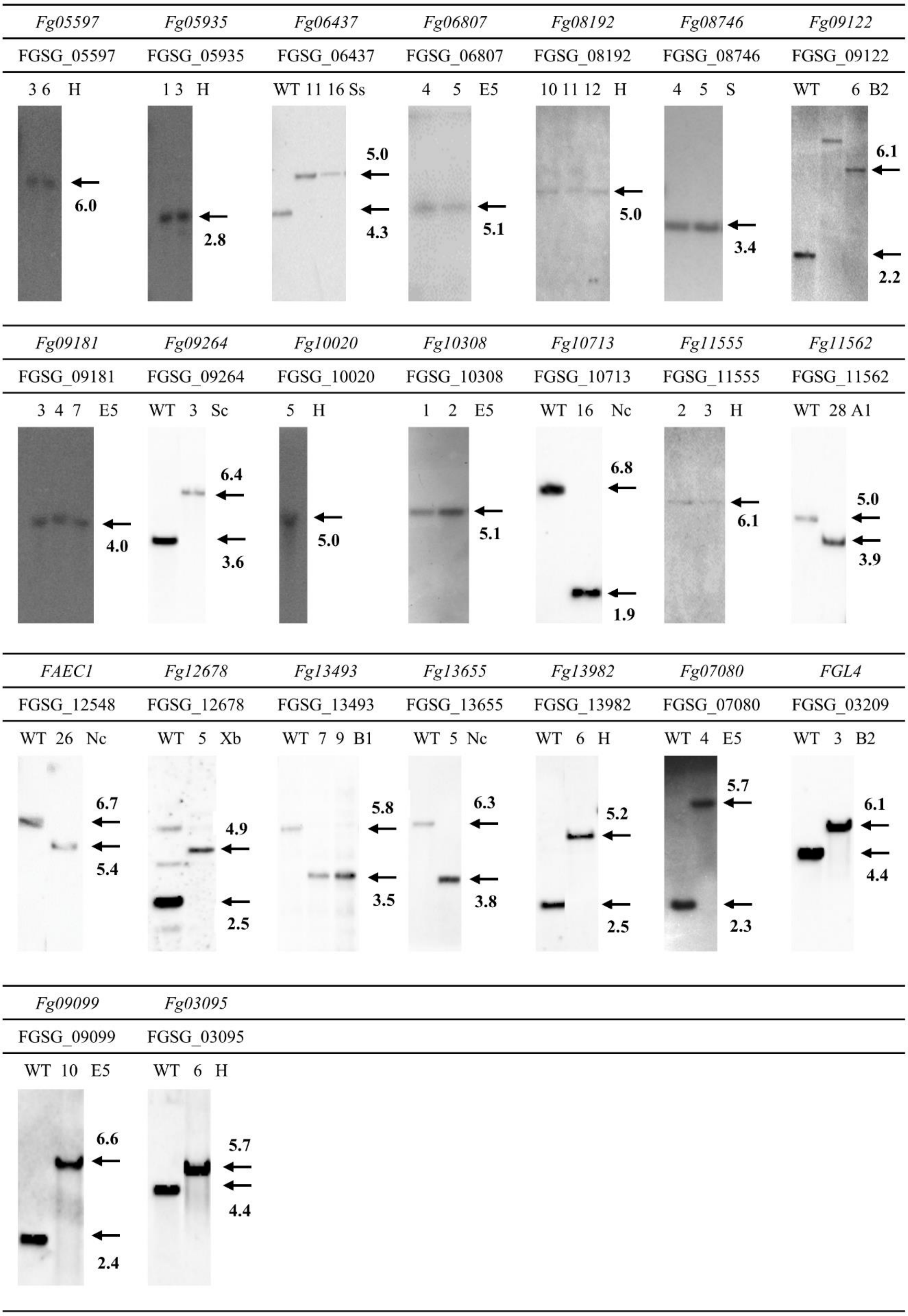

Fg05597
FGSG_05597
3 6 H
6.0
Fg05935
FGSG_05935
1 3 H
2.8
Fg06437
FGSG_06437
WT 11 16 Ss
5.0
4.3
Fg06807
FGSG_06807
4 5 E5
5.1
Fg08192
FGSG_08192
10 11 12 H
5.0
Fg08746
FGSG_08746
4 5 S
3.4
Fg09122
FGSG_09122
WT 6 B2
6.1
2.2
Fg09181
FGSG_09181
3 4 7 E5
4.0
Fg09264
FGSG_09264
WT 3 Sc
6.4
3.6
Fg10020
FGSG_10020
5 H
5.0
Fg10308
FGSG_10308
1 2 E5
5.1
Fg10713
FGSG_10713
WT 16 Nc
6.8
1.9
Fg11555
FGSG_11555
2 3 H
6.1
Fg11562
FGSG_11562
WT 28 A1
5.0
3.9
FAEC1
FGSG_12548
WT 26 Nc
6.7
5.4
Fg12678
FGSG_12678
WT 5 Xb
4.9
2.5
Fg13493
FGSG_13493
WT 7 9 B1
5.8
3.5
Fg13655
FGSG_13655
WT 5 Nc
6.3
3.8
Fg13982
FGSG_13982
WT 6 H
5.2
2.5
Fg07080
FGSG_07080
WT 4 E5
5.7
2.3
FGL4
FGSG_03209
WT 3 B2
6.1
4.4
Fg09099
FGSG_09099
WT 10 E5
6.6
2.4
Fg03095
FGSG_03095
WT 6 H
5.7
4.4

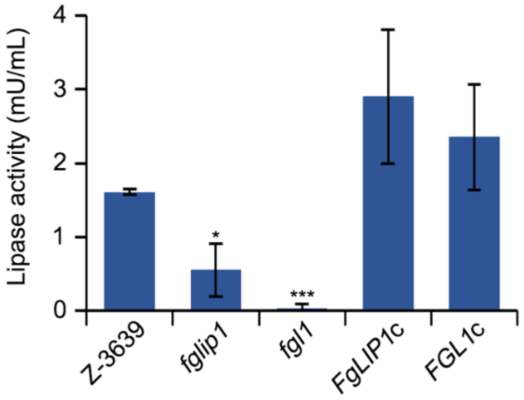

Figure S2. Triacylglycerol lipase activity of *fglip1*, *fgl1*, and complemented strains**.** Each strain was cultured for 12 h in the lipase-inducing medium and the culture supernatant was used for the analysis of triacylglycerol lipase activity. An asterisk indicates a significant difference (*P<0.01, ***P < 0.001) compared to the wild-type.

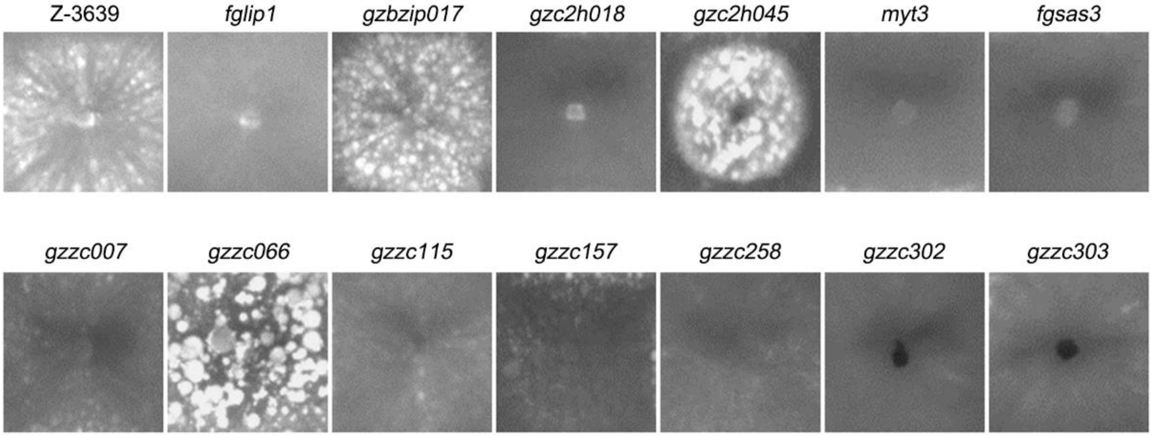

Figure S3. Lipase activity screening of TF mutants**.** Each TF mutant was inoculated on MM containing 1 % olive oil and 0.0005 % rhodamine B. The pictures were taken 2 days after inoculation under UV light.

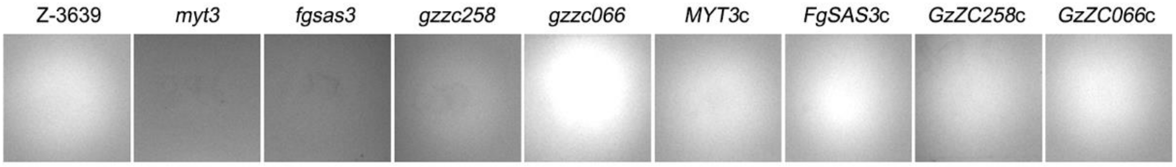

Figure S4. Lipase activity of four TF mutants and the complemented strains**.** Each strain was inoculated on MM containing 1% olive oil and 0.0005% rhodamine B. The pictures were taken 2 days after inoculation under UV light.

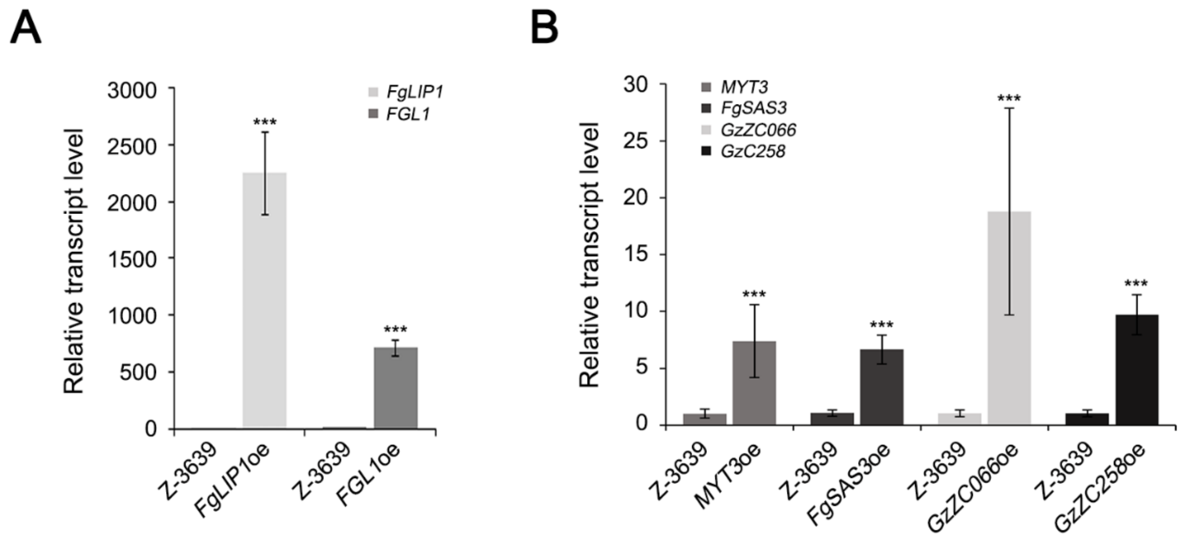

Figure S5. Overexpression of *FgLIP1*, *FGL1*, and four TF genes. (A and B) Overexpression of *FgLIP1*, *FGL1* (A), and *MYT3*, *FgSAS3*, *GzZC258*, and *GzZC066* (B) were confirmed via qRT-PCR. Total RNA of each strain was extracted 24 h after cultivation in CM, and the relative transcript level was quantified. Significant differences (***P < 0.001) in the relative transcript level of each gene in comparison with the wild-type are indicated with asterisks. The relative transcript abundances of the indicated gene in Z-3639 was arbitrarily set to 1.

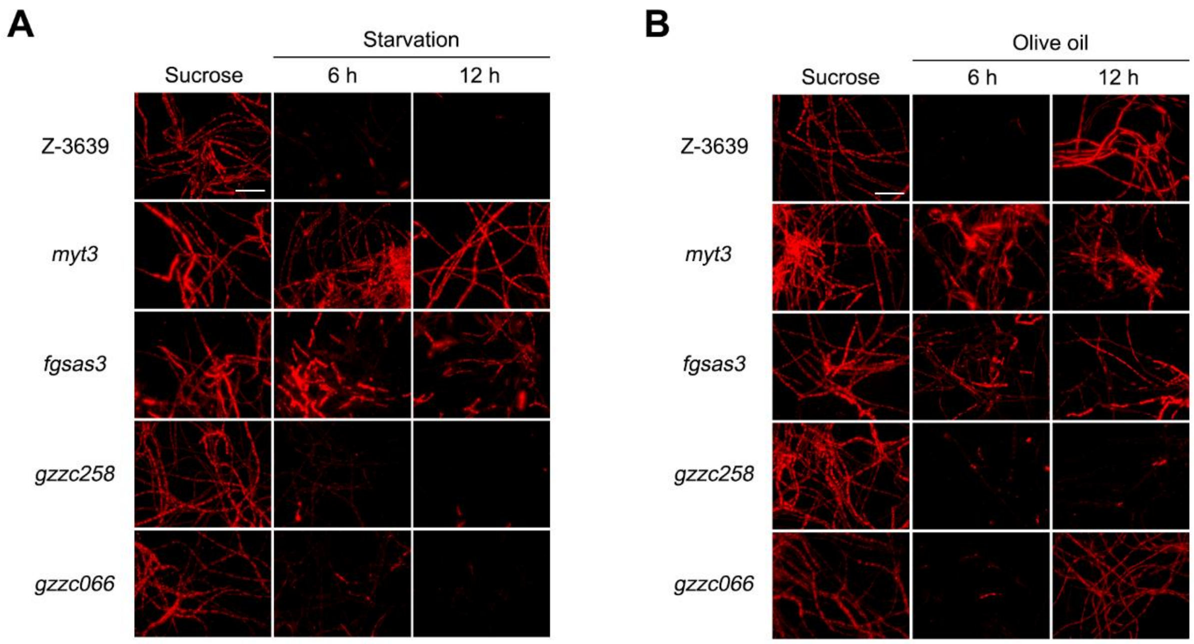

Figure S6. Visualization of intracellular lipid droplets in *F. graminearum* strains. (A and B) The mycelia were cultured in MM, MM without carbon source (A) and MM supplemented with 1 % olive oil as a sole carbon source (B) for indicated time points. The mycelia were stained with Nile red solution. Scale bar = 50 μm.

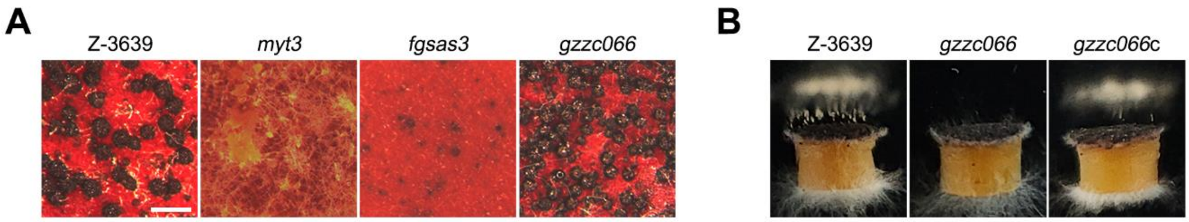

Figure S7. Sexual development of *F. graminearum* strains. (A) Perithecium formation. The perithecia were imaged 8 days after sexual induction. Scale bar = 500 μm. (B) Forcible ascospore discharge. White cloudy materials represent discharged ascospores. The pictures were taken 48 h after initiation of the assay.

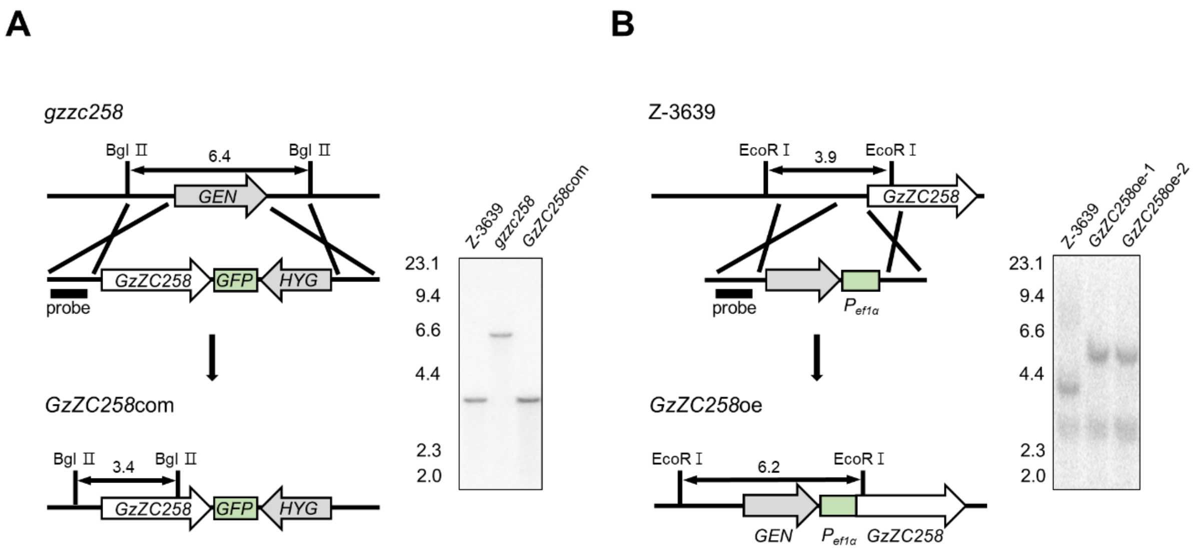

Figure S8. Genetic complementation and overexpression of *GzZC258.* Schematic illustrating the strategy for genetic complementation (A) and overexpression (B) of *GzZC258* (left panel). Southern blot analysis confirming genetic manipulations (right panel). Sizes of the DNA standards (kb) are indicated to the left of the blots.
